# New Cyclic Cystine Bridged Peptides from the Sponge *Suberites waedoensis*

**DOI:** 10.3390/md12052760

**Published:** 2014-05-12

**Authors:** Jinhaeng Song, Ju-eun Jeon, Tae Hyung Won, Chung J. Sim, Dong-Chan Oh, Ki-Bong Oh, Jongheon Shin

**Affiliations:** 1 Natural Products Research Institute, College of Pharmacy, Seoul National University, San 56-1, Sillim, Gwanak, Seoul 151-742, Korea; E-Mails: syoranzz@naver.com (J.S.); mirabn@snu.ac.kr (J.-e.J.); wth123@snu.ac.kr (T.H.W.); dongchanoh@snu.ac.kr (D.-C.O.); 2 Department of Biological Science, College of Life Science and Nano Technology, Hannam University, 461-6 Jeonmin, Yuseong, Daejeon 305-811, Korea; E-Mail: cjsim@hnu.kr; 3 Department of Agricultural Biotechnology, College of Agriculture and Life Science, Seoul National University, San 56-1, Sillim, Gwanak, Seoul 151-921, Korea

**Keywords:** *Suberites waedoensis*, cyclic cystine bridged peptides, chujamides, cytotoxicity, Na^+^/K^+^-ATPase

## Abstract

Two new peptides, chujamides A (**1**) and B (**2**), were isolated from the marine sponge *Suberites waedoensis*, which was collected from Korean waters. Based upon the results of the combined spectroscopic analyses, the structures of these compounds were determined to be proline-riched and cyclic cystine bridged dodeca- and undecapeptides. The absolute configurations of all amino acid residues were determined to be l by advanced Marfey’s analysis. The new compounds exhibited weak cytotoxicities against A549 and K562 cell-lines, and compound **2** also demonstrated moderate inhibitory activity against Na^+^/K^+^-ATPase.

## 1. Introduction

Sponges are widely recognized to be one of the most prolific sources of natural marine products with diverse biogenetic origins. Although peptides account for a relatively minor proportion of sponge-derived metabolites, several of these peptides possess highly unique chemical structures and potent bioactivities [1]. Among the recently reported peptides, noticeable examples include the kapakahines [2,3] from *Cribrochalina olemeda*, the neopetrosiamides [4] from *Neopetrosia* sp., the koshikamides and mutremdamide A [5,6] from *Theonella* spp., the yaku’amides [7] from *Ceratropsion* sp., and the solomonamides [8] from *Theonella swinhoei*. These compounds possess unusual amino acid residues, uncommon linkages and/or significant bioactivities.

**Figure 1:**
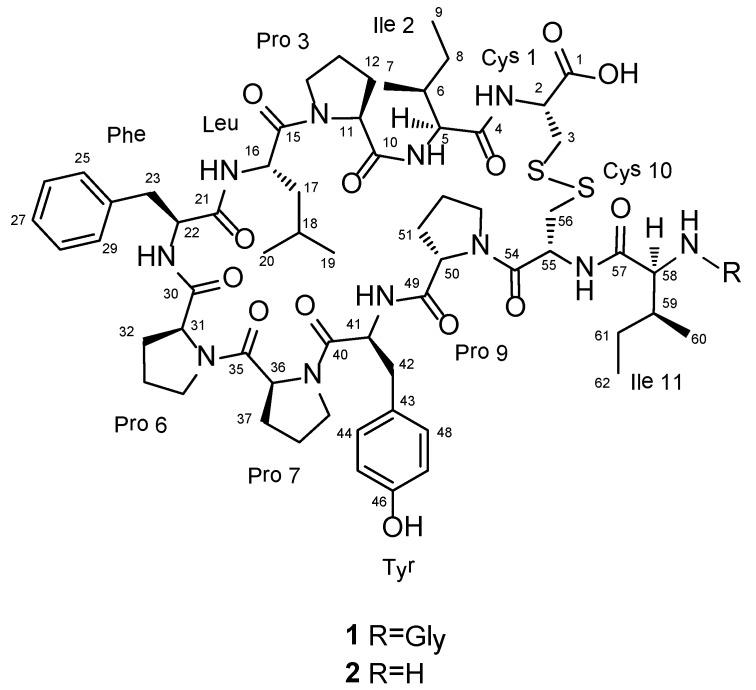
Structures of compounds **1** and **2**.

In our search for bioactive metabolites from sponges found in Korean waters, we recently reported the structural determination of gombamide A, a cyclic thiohexapeptide from *Clathria gombawuiensis* [9]. This highly modified peptide exhibited cytotoxicity against the A549 and K562 cell lines as well as inhibitory activity against Na^+^/K^+^-ATPase. In our continuing search for bioactive metabolites in sponges, we report here the isolation and structural determinations of chujamides A (**1**) and B (**2**), new cyclic cystine bridged peptides from the sponge *Suberites* *waedoensis* (Figure 1). These proline-rich dodeca- and undeca-peptides exhibited weak cytotoxicities, and compound **2** demonstrated moderate inhibitory activity against Na^+^/K^+^-ATPase.

## 2. Results and Discussion

**Figure 2:**
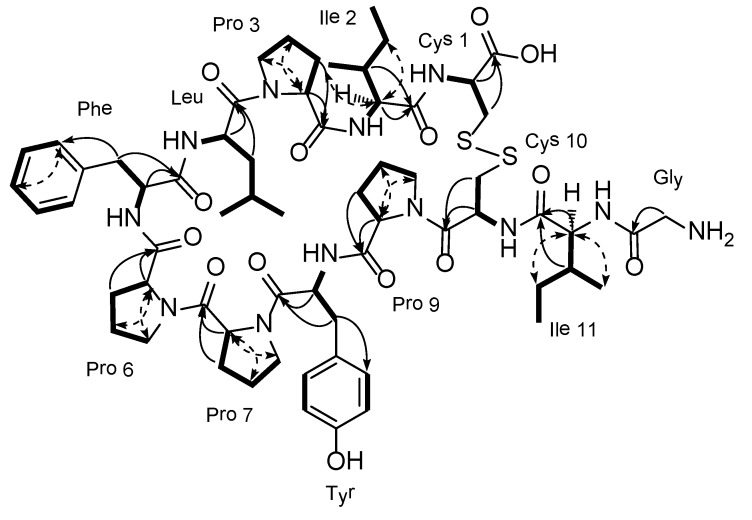
Key correlations within the individual amino acid residues of the COSY (**bold line**), TOCSY (**dashed arrow**), and gHMBC (**solid arrow**) experiments for chujamide A.

The molecular formula of chujamide A (**1**) was determined to be C_64_H_92_N_12_O_14_S_2_ by HRFABMS analysis. Evidence of the peptide nature of this compound was given by the presence of several carbonyl and methine carbons in the regions of δ_C_ 177-169 and 65-50, respectively, in the ^13^C NMR data. The structures of individual amino acid residues were determined by a combination of ^1^H COSY, TOCSY, HSQC, and HMBC experiments (See Supplementary Information, Figures S1–S12), which led to the identification of four prolines (Pro), two cysteines (Cys), two isoleucines (Ile), and one unit each of glycine (Gly), leucine (Leu), phenylalanine (Phe), and tyrosine (Tyr). Notably, due to poor resolution in DMSO-*d*_6_ and pyridine-*d*_5_, the 2-D NMR work was first performed in a MeOH-*d*_4_ solution to evaluate the protons attached to the carbon atoms. These data were supported by data concerning the amide protons obtained in MeOH-*d*_3_ solution. In this manner, all of the protons and carbon atoms, including the carbonyl carbon atoms, of each amino acid residue in **1** were assigned (Table 1 and Figure 2).

**Table 1:** ^13^C (150 MHz) and ^1^H (600 MHz) NMR assignments for chujamides A and B in MeOH-*d*_4_ and MeOH-*d*_3_^a^.

A. A. unit	Position	chujamide A		chujamide B	
		δ_C_	δ_H_ (*J* in Hz)	δ_C_	δ_H_ (*J* in Hz)
Cys 1	1	176.7, C		176.9, C	
	2	60.1, CH	4.71, dd (11.4, 2.4)	60.3, CH	4.71, dd (11.4, 2.4)
	3	43.0, CH_2_	3.45, m	43.0, CH_2_	3.47, m
			2.55, m		2.58, m
	2-NH ^a^		7.81, d (9.6)		8.50, bs
Ile 2	4	172.4, C		173.6, C	
	5	59.1, CH	4.82, d (3.6)	59.2, CH	4.82, m
	6	37.7, CH	2.34, m	38.0, CH	2.32, m
	7	16.1, CH_3_	0.84, d (6.6)	16.1, CH_3_	0.83, d (6.6)
	8	25.2, CH_2_	1.54, m	25.4, CH_2_	1.55, m
			0.94, m		0.95, m
	9	12.2 CH_3_	0.90, t (6.6)	12.2, CH_3_	0.90, t (6.6)
	5-NH ^a^		7.52, d (10.2)		7.67, bs
Pro 3	10	174.6, C		173.4, C	
	11	61.9, CH	5.24, d (7.8)	61.5, CH	5.25, m
	12	26.8, CH_2_	2.53, m	27.0, CH_2_	2.48, m
			1.72, m		1.73, m
	13	26.2, CH_2_	2.15, m	26.3, CH_2_	2.13, m
			1.97, m		1.93, m
	14	48.3, CH_2_	3.81, m	48.3, CH_2_	3.80, m
			3.63, dd (8.4, 8.4)		3.64, m
Leu	15	176.4, C		176.5, C	
	16	51.7, CH	4.50, dd (12.0, 1.8)	51.6, CH	4.51, dd (12.0, 1.8)
	17	41.4, CH_2_	1.73, m	41.6, CH_2_	1.72, m
			1.40, m		1.34, m
	18	25.9, CH	1.71, m	26.0, CH	1.70, m
	19	20.5, CH_3_	0.97, d (6.6)	20.5, CH_3_	0.97, d (6.6)
	20	24.0, CH_3_	0.99, d (6.6)	24.0, CH_3_	0.98, d (6.6)
	16-NH ^a^		7.78, d (4.8)		7.77, d (4.8)
Phe	21	173.7, C		173.7, C	
	22	56.3, CH	4.46, dd (12.0, 4.8)	56.4, CH	4.46, dd (12.0, 4.8)
	23	33.8, CH_2_	3.41, m	33.9, CH_2_	3.41, m
			3.01, m		3.00, m
	24	140.2, C		140.3, C	
	25/29	131.1, CH	7.27, dd (7.2, 1.8)	131.2, CH	7.27, dd (7.2, 1.8)
	26/28	129.1, CH	7.14, m	129.1, CH	7.14, m
	27	127.3, CH	7.14, m	127.3, CH	7.14, m
	22-NH ^a^		8.79, d (7.2)		8.80, d (7.2)
Pro 6	30	171.9, C		171.9, C	
	31	62.5, CH	4.17, d (7.8)	62.5, CH	4.15, d (7.8)
	32	30.3, CH_2_	2.55, m	30.3, CH_2_	2.54, m
			1.97, m		1.99, m
	33	23.1, CH_2_	1.97, m	23.1,CH_2_	1.90, m
			1.60, m		1.62, m
	34	47.8, CH_2_	3.50, m	47.8, CH_2_	3.50, m
			3.44, m		3.44, m
Pro 7	35	172.0, C		172.1, C	
	36	59.9, CH	2.99, m	59.9, CH	2.99, m
	37	29.4, CH_2_	2.00, m	29.4, CH_2_	2.01, m
			1.61, m		1.62, m
	38	26.0, CH_2_	2.00, m	25.9, CH_2_	2.01, m
			1.94, m		1.93, m
	39	48.8, CH_2_	3.61, m	48.7, CH_2_	3.61, m
			3.53, m		3.53, m
Tyr	40	171.2, C		171.3, C	
	41	52.7, CH	4.85, t (6.0)	52.6, CH	4.84, t (6.0)
	42	36.9, CH_2_	3.00, m	37.0, CH_2_	3.01, m
			2.74, dd (14.4, 6.0)		2.75, m
	43	127.2, C		127.4, C	
	44/48	132.1, CH	6.98, d (8.4)	132.1, CH	6.98, d (8.4)
	45/47	116.1, CH	6.71, d (8.4)	116.3, CH	6.70, d (8.4)
	46	157.7, C		157.7, C	
	41-NH ^a^		7.05, d (7.2)		7.11, m
Pro 9	49	172.5, C		172.4, C	
	50	62.2, CH	4.68, d (8.4)	62.2, CH	4.69, d (8.4)
	51	28.7, CH_2_	2.32, m	28.6, CH_2_	2.32, m
			2.00, m		2.00, m
	52	25.7, CH_2_	2.09, m	25.7, CH_2_	2.09, m
			1.80, m		1.80, m
	53	48.6, CH_2_	3.87, m	48.6, CH_2_	3.87, m
			3.73, m		3.73, m
Cys 10	54	172.7, C		172.7, C	
	55	51.2, CH	4.77, dd (12.0, 2.4)	51.5, CH	4.76, ddd (12.0, 2.4, 2.4)
	56	40.3, CH_2_	3.09, m	40.5, CH_2_	3.08, m
			2.77, dd (14.4, 3.0)		2.73, m
	55-NH ^a^		8.58, d (4.8)		8.27, bs
Ile 11	57	174.3, C		174.3, C	
	58	59.7, CH	4.01, d (10.2)	60.0, CH	3.95, m
	59	37.2, CH	1.72, m	37.2, CH	1.72, m
	60	15.8, CH_3_	0.93, d (6.6)	15.8, CH_3_	0.93, d (6.6)
	61	26.7, CH_2_	1.55, m	26.8, CH_2_	1.55, m
			1.18, m		1.17, m
	62	10.7, CH_3_	0.86, t (6.6)	10.8, CH_3_	0.86, t (6.6)
	58-NH ^a^		8.73, d (6.6)		
Gly	63	169.4, C			
	64	41.8, CH_2_	3.57, m		
			3.55, m		

^a^ Amide protons were observed in the spectra obtained in MeOH-*d*_3_ solution; A. A.: Amino Acid.

Confirmation of the amino acid residues as well as the absolute configuration of each residue in compound **1** was accomplished by advanced Marfey’s analysis [10,11]. After the acid hydrolysis of **1**, ESI-LC/MS analysis of the hydrolysate adducts with l-FDAA (1-fluoro-2-4-dinitrophenyl-5-l-alanine amide) and d-FDAA clearly confirmed the NMR-based amino acid residue assignments. Comparison of the ESI-LC/MS retention times of the l- and d-FDAA-derivatized hydrolysates allowed the assignment of the l configuration to all of the amino acid residues. In addition, the configurations of the β-carbons of the two l-Ile residues were both also assigned to be *S* by the co-injection of both l- and l-*allo*-Ile with the hydrolysates during ESI-LC/MS analysis.

**Figure 3:**
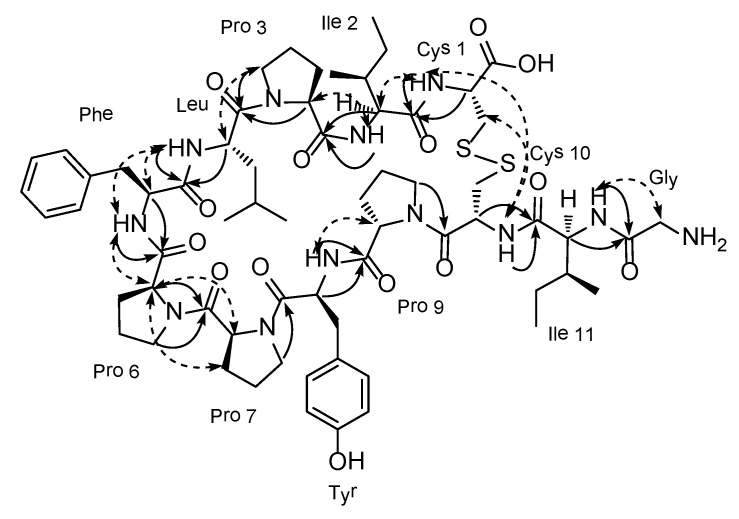
Key correlations between the neighboring amino acid residues of chujamide A from gHMBC (**solid arrow**) and ROESY (**dashed arrow**) experiments.

Given the identity of the amino acid residues, the structural construction of **1** was accomplished by a combination of HMBC and NOESY experiments (Figure 3). That is, the long-range correlations of the H-2 (δ_H_ 4.71) and 2-NH (δ_H_ 7.81) protons with the C-4 carbonyl carbon (δ_C_ 172.4) in the HMBC data suggested a peptide linkage between a Cys (Cys 1) and an Ile (Ile 2) that was supported by the NOESY cross peak at 2-NH/H-5. Similarly, the placement of a Pro (Pro 3) at the other end of this Ile was supported by the HMBC correlations at H-5/C-10 and 5-NH/C-10 as well as the NOESY cross peak at 5-NH/H-11. In this manner, the linear assembly of twelve amino acid residues connected to each other by peptide bonds was unambiguously identified as Cys 1-Ile 2-Pro 3-Leu-Phe-Pro 6-Pro 7-Tyr-Pro 9-Cys 10-Ile 11-Gly.

Due to the lack of suitable carbons and protons within a two or three bond distance, the disulfide linkage between Cys-1 and Cys-10, inherent in the molecular formula as an additional degree of unsaturation, was not supported by the HMBC data but was indicated by the NOESY cross-peaks at 2-NH/55-NH and H-3/55-NH. The presence of a disulfide linkage between the cysteine residues was also supported by the chemical shifts of the C_β_ carbons at δ_C_ 40.3 and 40.0, which were similar to those of other peptides possessing cysteines with a similar disulfide linkage [12]. For comparison, the shifts of free cysteine and cysteic acid were approximately δ_C_ 25.5 and 50, respectively [13,14]. Thus, chujamide A (**1**) was determined to be a novel cyclic cystine bridged dodecapeptide.

A related compound, chujamide B (**2**), was also isolated as an amorphous solid. Although its molecular formula was not directly determined from the high-resolution MS analysis, the MALDITOF-MS data, in conjunction with the ^1^H and ^13^C NMR data, established the molecular formula to be C_62_H_89_N_11_O_13_S_2_ [4]. The spectroscopic data of this compound were highly reminiscent of the data of **1**, and the loss of signals for a carbonyl and a methylene were the most noticeable differences in both the ^1^H and ^13^C NMR data. These spectroscopic differences were readily accounted for by the loss of a terminal Gly residue, which was confirmed by combined 2-D NMR experiments in which the same proton-proton and proton-carbon correlations as those in **1** were obtained for the entire cyclic portion (See Supplementary Information, Figures S13–S24). Thus, chujamide B (**2**) was determined to be a cyclic cystine bridged undecapeptide.

Cyclic peptides with disulfide linkages have been rarely found in sponges, as the microcionamides from *Clathria abietina* [12], the neopetrosiamides from *Neopetrosia* sp. [4], asteropsin A from *Asteropus* sp. [15], and the recently reported gombamide A from *Clathria gombawuiensis* [9] are the only examples in the literature, which confirms the scarcity of these peptides. A literature survey also revealed that previous works on the peptides from sponges of the genus *Suberites* only yielded geodiamolides and serangamides [16], cyclic and linear lipotripeptides related to jasplakinolides [17] (=jaspamide [18]) whose frameworks differ significantly from those of the chujamides.

In our bioactivity measurements, chujamides A (**1**) and B (**2**) exhibited weak cytotoxicities toward the K562 and A549 cell-lines. The LC_50_ values were 37.0 and 10.1 µM for compound **1** and were 55.6 and 26.4 µM for compound **2**, respectively (the LC_50_ values of doxorubicin were 1.5 and 1.3 µM, respectively). Chujamide B also moderately inhibited the action of Na^+^/K^+^-ATPase with an IC_50_ value of 17.2 µM (the IC_50_ value of ouabain was 6.1 µM). However, these compounds were inactive (MIC > 100 mM) against strains of Gram-positive and Gram-negative bacteria and pathogenic fungi [19].

In summary, two cyclic cystine bridged peptides rich in prolines, chujamides A (**1**) and B (**2**), were isolated from the Korean sponge *Suberites waedoensis* and were structurally elucidated by combining spectroscopic and Marfey’s analyses. These compounds exhibited weak cytotoxicities, and compound **2** demonstrated moderate inhibition against Na^+^/K^+^-ATPase.

## 3. Experimental Section

### 3.1. General Experimental Procedures

Optical rotations were measured on a JASCO P-1020 polarimeter using a 1-cm cell. UV spectra were recorded on a Hitachi U-3010 spectrophotometer, and IR spectra were recorded on a JASCO 300E FT-IR spectrometer. NMR spectra were recorded in MeOH-*d*_4_ and MeOH-*d*_3_ containing Me_4_Si as an internal standard on a Bruker Avance 600 spectrometer. Proton and carbon NMR spectra were measured at 600 and 150 MHz (**1** and **2**), respectively. Mass spectrometric data were obtained at the Korea Basic Science Institute (Daegu, Korea) and were acquired using a JEOL JMS 700 mass spectrometer with meta-nitrobenzyl alcohol (NBA) as the matrix for the FABMS. MALDI-TOF was provided by National Center for Inter-University Research Facilities (Seoul, Korea) and was acquired using a Voyager-DE™ STR Biospectrometry Workstation (Foster City, CA, USA) for compound **2**. Low-resolution ESIMS data were recorded on an Agilent Technologies 6130 quadrupole mass spectrometer with an Agilent Technologies 1200 series HPLC (Agilent Technologies, Santa Clara, CA, USA). HPLC was performed on a SpectraSystem p2000 equipped with a SpectraSystem RI-150 (Thermo, Waltham, MA, USA) refractive index detector. All of the solvents were of spectroscopic grade or were distilled in glass prior to use.

### 3.2. Animal Materials

Specimens of *Suberites waedoensis* (voucher collection number 12CH-1) were collected by hand using scuba equipment off the shore of Chuja Island, Korea at a depth of 25 m during 8–11 October 2012. The sponge was cushion shaped, had a red color in life, and measured 12 × 10 cm with a thickness of 3 cm. The surface was lightly wrinkled but smooth, and the texture was elastic. The skeleton was small and had large tightly arranged tylostyles (200 − 400 × 5 μm and 600 − 900 × 10 − 16 μm). These morphological features agreed well with those reported in the literature [20]. A voucher specimen (registry no. spo 71) was deposited at the Natural History Museum, Hannam University, Korea, under the curatorship of C.J. Sim.

### 3.3. Extraction and Isolation

The freshly collected specimens were frozen immediately and kept at −25 °C until used for the chemical investigation. The freeze-dried sponge was sliced and repeatedly extracted with MeOH (3 × 3 L) and CH_2_Cl_2_ (3 × 3 L). The combined organic extract (354.5 g) was partitioned between H_2_O (210.8 g) and *n*-BuOH (140.7 g), and the latter was then repartitioned between *n*-hexane (120.1 g) and H_2_O–MeOH (15:85, 18.5 g). An aliquot (9.2 g) of the H_2_O–MeOH layer from the solvent partitioning was subjected to reversed-phase vacuum flash chromatography using a sequential mixture of H_2_O and MeOH (six fractions in the gradient, H_2_O–MeOH, from 50:50 to 0:100), acetone, and finally EtOAc as the eluents.

Based on the results of the ^1^H NMR and the cytotoxicity analyses, the fraction that eluted with H_2_O-MeOH (30:70; 0.23 g) was selected for separation. This fraction was separated by semi-preparative reversed-phase HPLC (YMC ODS-A column, 10 × 250 mm, H_2_O–MeOH, 40:60) to yield, in order of their elution, compounds **2** and **1**. The final purification of the individual compounds was then accomplished by reversed-phase HPLC (YMC-Pack CN column, 4.6 × 250 mm, H_2_O–MeOH, and 50:50 for **1** and YMC ODS-A column, 4.6 × 250 mm, and H_2_O–MeOH, 45:55 for **2**). Compound **1** was also isolated from the flash chromatographic fraction eluted with H_2_O–MeOH (20:80; 0.29 g) using the same HPLC conditions. The purified metabolites were isolated in the following amounts: 27.0 mg for **1** and 4.0 mg for **2**.

Chujamide A (**1**): white, amorphous solid, 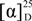 −15 (*c* 0.50, MeOH); UV (MeOH) λ_max_ (log ε) 210 (4.40), 227 (4.14), 277 (3.16) nm; IR (ZnSe) *v*_max_ 3300, 2958, 1655 cm^−1^; ^1^H and ^13^C NMR data, see Table 1; HRFABMS *m/z* 1317.6371 [M + H]^+^ (calcd for C_64_H_93_N_12_O_14_S_2_, 1317.6367).

Chujamide B (**2**): white, amorphous solid, 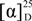 −52 (*c* 0.45, MeOH); UV (MeOH) λ_max_ (log ε) 210 (4.41), 227 (4.10), 276 (3.16) nm; IR (ZnSe) *v*_max_ 3308, 2956, 1637 cm^−1^; ^1^H and ^13^C NMR data, see Table 1; MALDITOF-MS *m/z* 1260 [M]^+^ (calcd for C_62_H_90_N_11_O_13_S_2_, 1260).

### 3.4. Advanced Marfey’s Analysis of Compound 1

Compound **1** (1.0 mg) was dissolved in 0.5 mL of 6 N HCl and heated at 110 °C for 15 h. This solution was evaporated, and traces of HCl were removed by repeatedly drying the compound under vacuum with distilled water. To the divided hydrolysate (0.5 mg), 100 μL of 1 N NaHCO_3_ and 50 μL of 1% l- or d-FDAA in acetone were added. The mixture was stirred at 70 °C for 1 h. After the reaction was quenched by the addition of 50 μL of 2 N HCl, the mixture was analyzed by ESI-LC/MS (YMC ODS-A column, 5 μm, 4.6 × 100 mm, H_2_O-MeCN gradient (80:20 to 30:70 in 40 min, v/v), 0.7 mL/min flow rate, UV detector, 360 nm) to assign the chirality of the amino acids. The retention times of the l- and d-FDAA-derivatized hydrolysates were 13.5 min and 14.4 min for l-Pro, 15.3 min and 16.5 min for l-Tyr, 16.9 min and 18.9 min for l-Cys, 20.6 min and 24.0 min for l-Ile, 21.3 min and 24.5 min for l-Leu, and 21.4 min and 23.9 min for l-Phe, respectively. Thus, all of the amino acids appeared to be l-amino acids.

### 3.5. Analysis of the Configuration of the l-Ile Residue in Compound 1

The absolute configuration of the β-carbon of l-Ile moiety was identified by an ESI-LC/MS experiment with the l-FDAA derivatives of standard l-*allo*-Ile and l-Ile. Under the given chromatographic conditions (YMC ODS-A column, 5 μm, 4.6 × 250 mm, H_2_O-MeCN gradient (80:20 to 30:70 in 40 min, v/v), 0.7 mL/min flow rate, UV detector, 360 nm), the l-FDAA derivatives of authentic l-*allo*-Ile and l-Ile had retention times of 38.349 and 37.845 min, respectively. In addition, co-injection with the l-FDAA derivative of the hydrolysate of the l-Ile moiety showed one single peak of l-Ile-l-FDAA at 37.845 min. Thus, the absolute configurations of both l-Ile moieties in compound **1** were determined to be *S*.

### 3.6. Biological Assays

The cytotoxicity assays were performed in accord with literature protocols [21,22]. The Na^+^/K^+^-ATPase inhibition assay was performed according to the previously described method [23].

## 4. Conclusions

Two new peptides, chujamides A (**1**) and B (**2**), were isolated from the marine sponge *Suberites waedoensis*, from Korea. These compounds were structurally classified to a group of the proline-riched and cyclic cystine bridged dodeca- and undecapeptides. These compounds exhibited weak cytotoxicity and moderate inhibition against Na^+^/K^+^-ATPase (**2**).

## Supplementary Files

Supplementary File 1 Supplementary Information (PDF, 2809 KB)
