# Supplementary material for: New Cyclic Cystine Bridged Peptides from the Sponge Suberites waedoensis
Source: Mar Drugs. 2014 May 12;12(5):2760–70. doi: 10.3390/md12052760 (PMC4052314; doi:10.3390/md12052760)
Supplement: Supplementary File 1 — Supplementary Information (PDF, 2809 KB) [file marinedrugs-12-02760-s001.pdf]

Supplementary Information

Table of Content

I. Compound **1**

Figure S1. The ^{1}H NMR (600 MHz, MeOH-d_4) spectrum of compound **1**
Figure S2. The ^{13}C NMR (150 MHz, MeOH-d_4) spectrum of compound **1**
Figure S3. The *g*HSQC (600 MHz, MeOH-d_4) spectrum of compound **1**
Figure S4. The *g*HMBC (600 MHz, MeOH-d_4) spectrum of compound **1**
Figure S5. The TOCSY (600 MHz, MeOH-d_4) spectrum of compound **1**
Figure S6. The COSY (600 MHz, MeOH-d_4) spectrum of compound **1**
Figure S7. The ROESY (600 MHz, MeOH-d_4) spectrum of compound **1**
Figure S8. The ^{1}H NMR (600 MHz, MeOH-d_3) spectrum of compound **1**
Figure S9. The *g*HMBC (600 MHz, MeOH-d_3) spectrum of compound **1**
Figure S10. The TOCSY (600 MHz, MeOH-d_3) spectrum of compound **1**
Figure S11. The COSY (600 MHz, MeOH-d_3) spectrum of compound **1**
Figure S12. The ROESY (600 MHz, MeOH-d_3) spectrum of compound **1**

II. Compound **2**

Figure S13. The ^{1}H NMR (600 MHz, MeOH-d_4) spectrum of compound **2**
Figure S14. The ^{13}C NMR (150 MHz, MeOH-d_4) spectrum of compound **2**
Figure S15. The *g*HSQC (600 MHz, MeOH-d_4) spectrum of compound **2**
Figure S16. The *g*HMBC (600 MHz, MeOH-d_4) spectrum of compound **2**
Figure S17. The TOCSY (600 MHz, MeOH-d_4) spectrum of compound **2**
Figure S18. The COSY (600 MHz, MeOH-d_4) spectrum of compound **2**
Figure S19. The ROESY (600 MHz, MeOH-d_4) spectrum of compound **2**
Figure S20. The ^{1}H NMR (600 MHz, MeOH-d_3) spectrum of compound **2**
Figure S21. The *g*HMBC (600 MHz, MeOH-d_3) spectrum of compound **2**
Figure S22. The TOCSY (600 MHz, MeOH-d_3) spectrum of compound **2**
Figure S23. The COSY (600 MHz, MeOH-d_3) spectrum of compound **2**
Figure S24. The ROESY (600 MHz, MeOH-d_3) spectrum of compound **2**

Figure S1. The ^{1}H NMR (600 MHz, MeOH-d_4) spectrum of compound **1**.

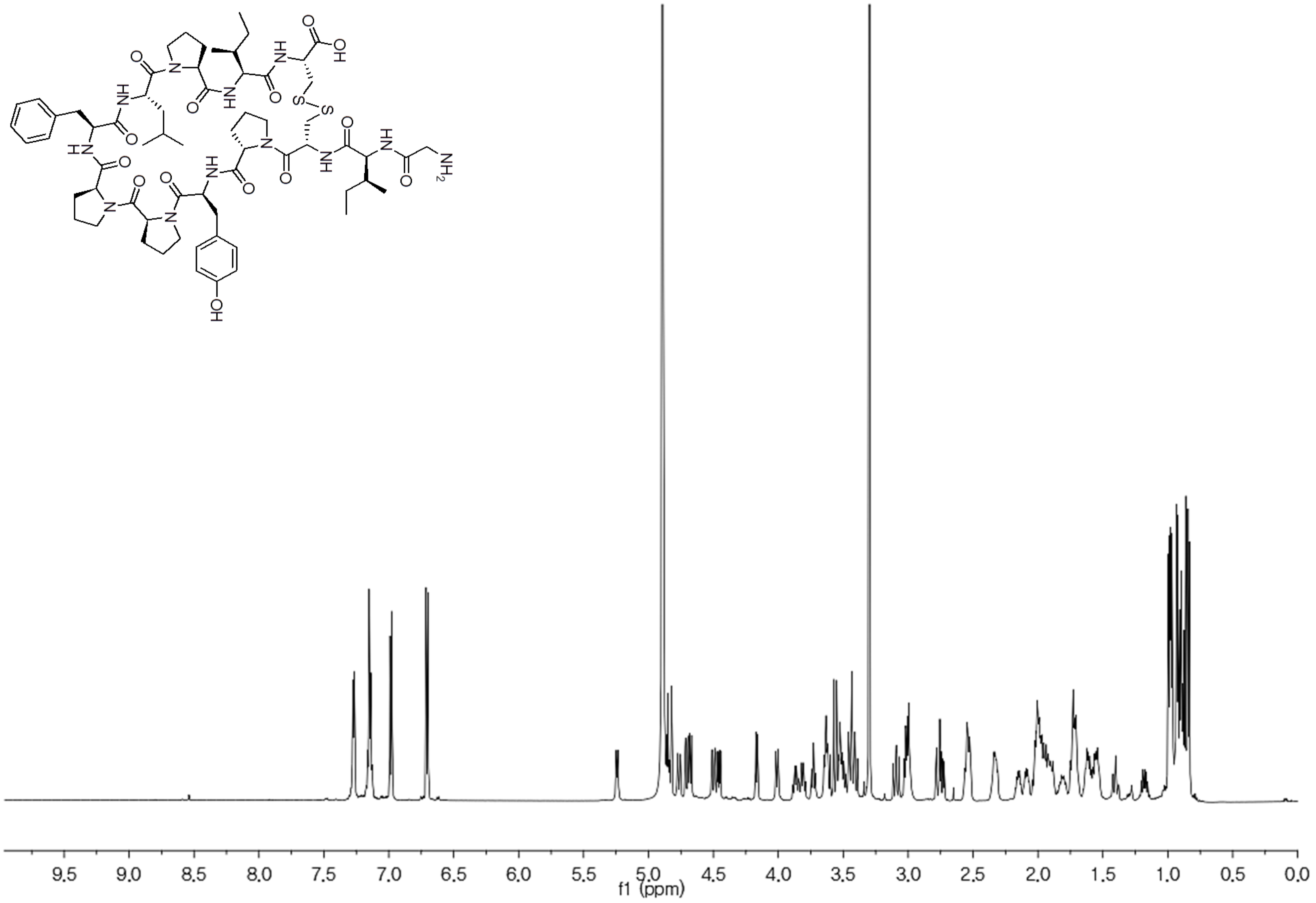

Figure S2. The ^{13}C NMR (150 MHz, MeOH-d_4) spectrum of compound **1**.

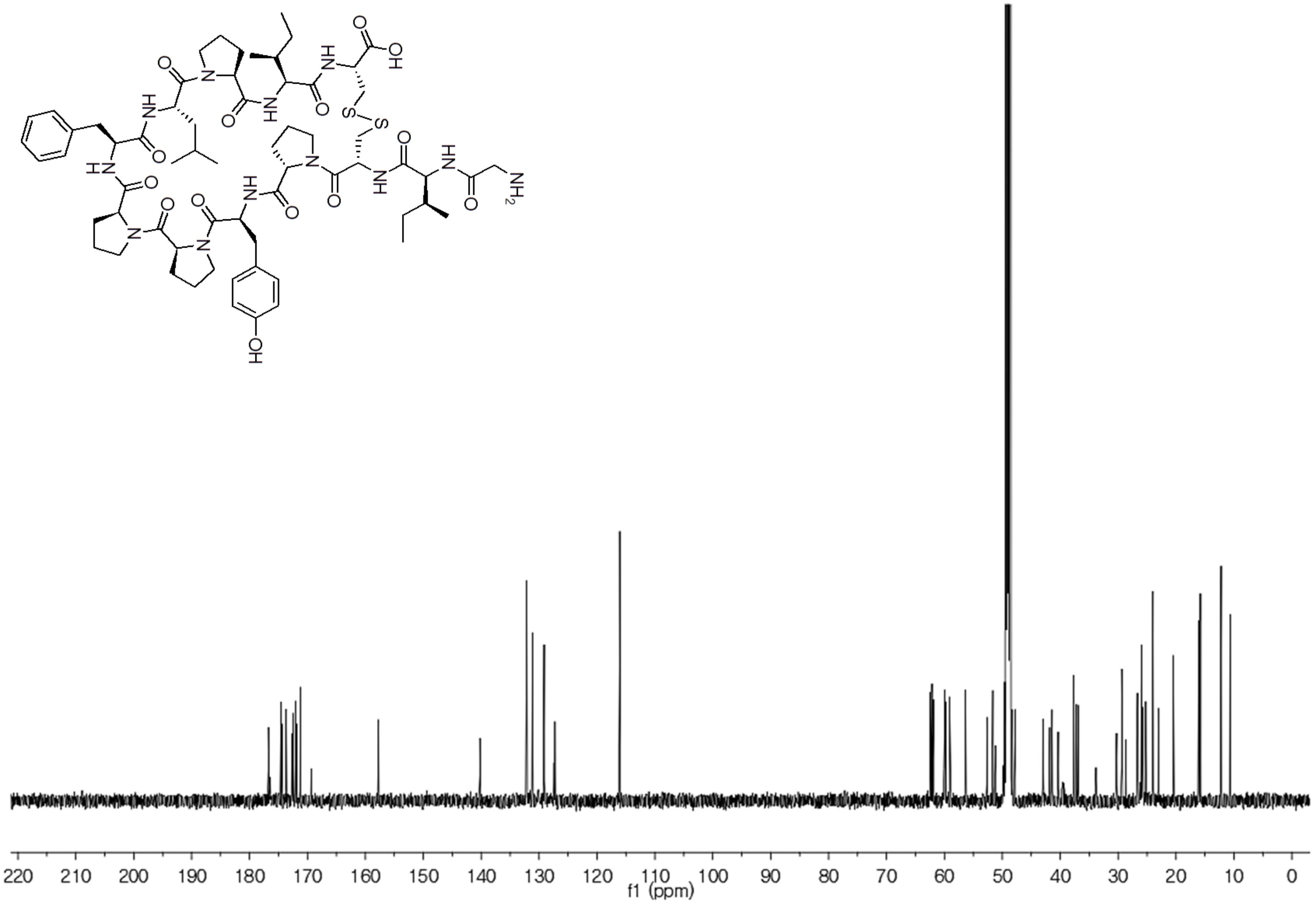

Figure S3. The *g*HSQC (600 MHz, MeOH-d_4) spectrum of compound **1**.

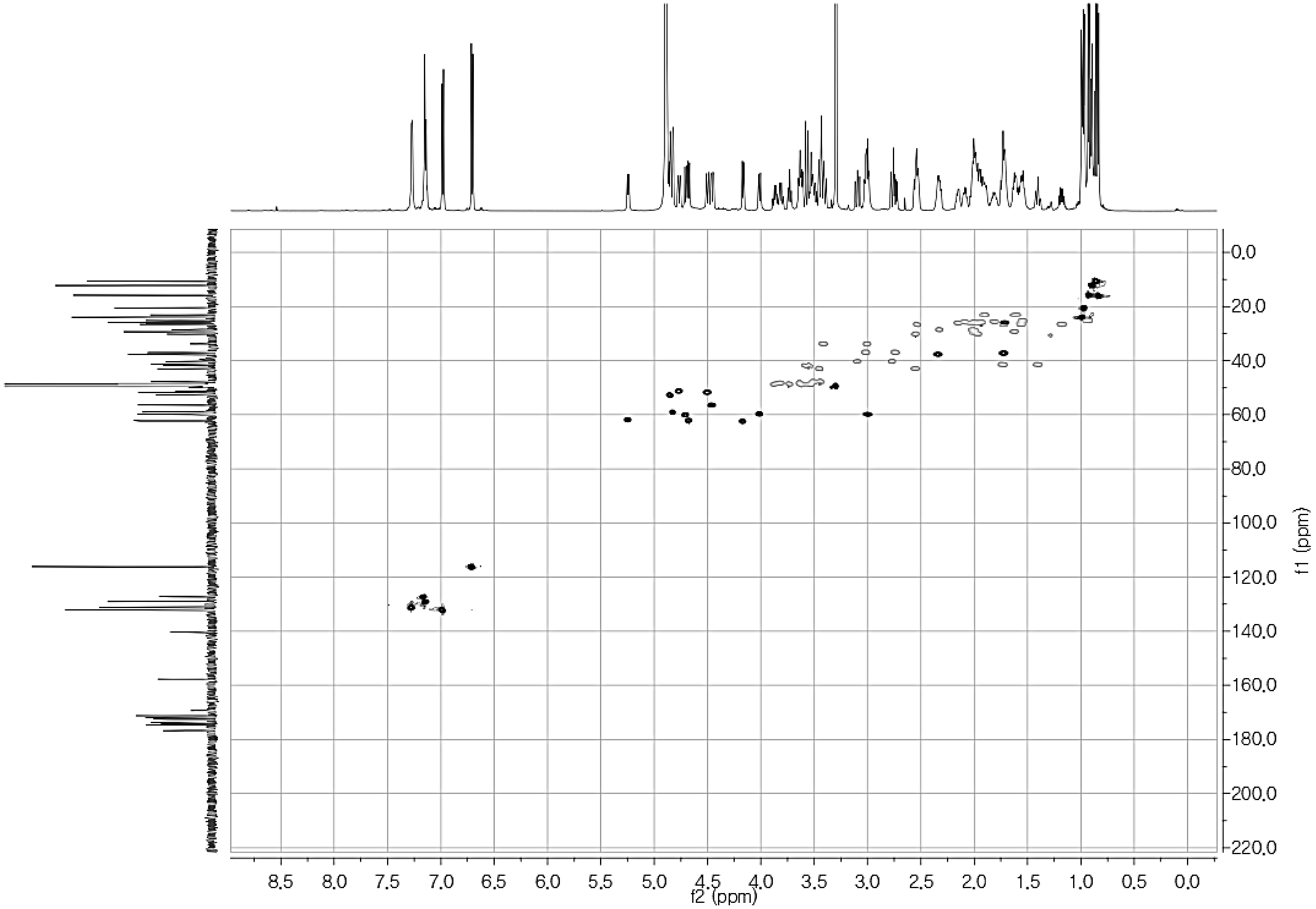

Figure S4. The *g*HMBC (600 MHz, MeOH-d_4) spectrum of compound **1**.

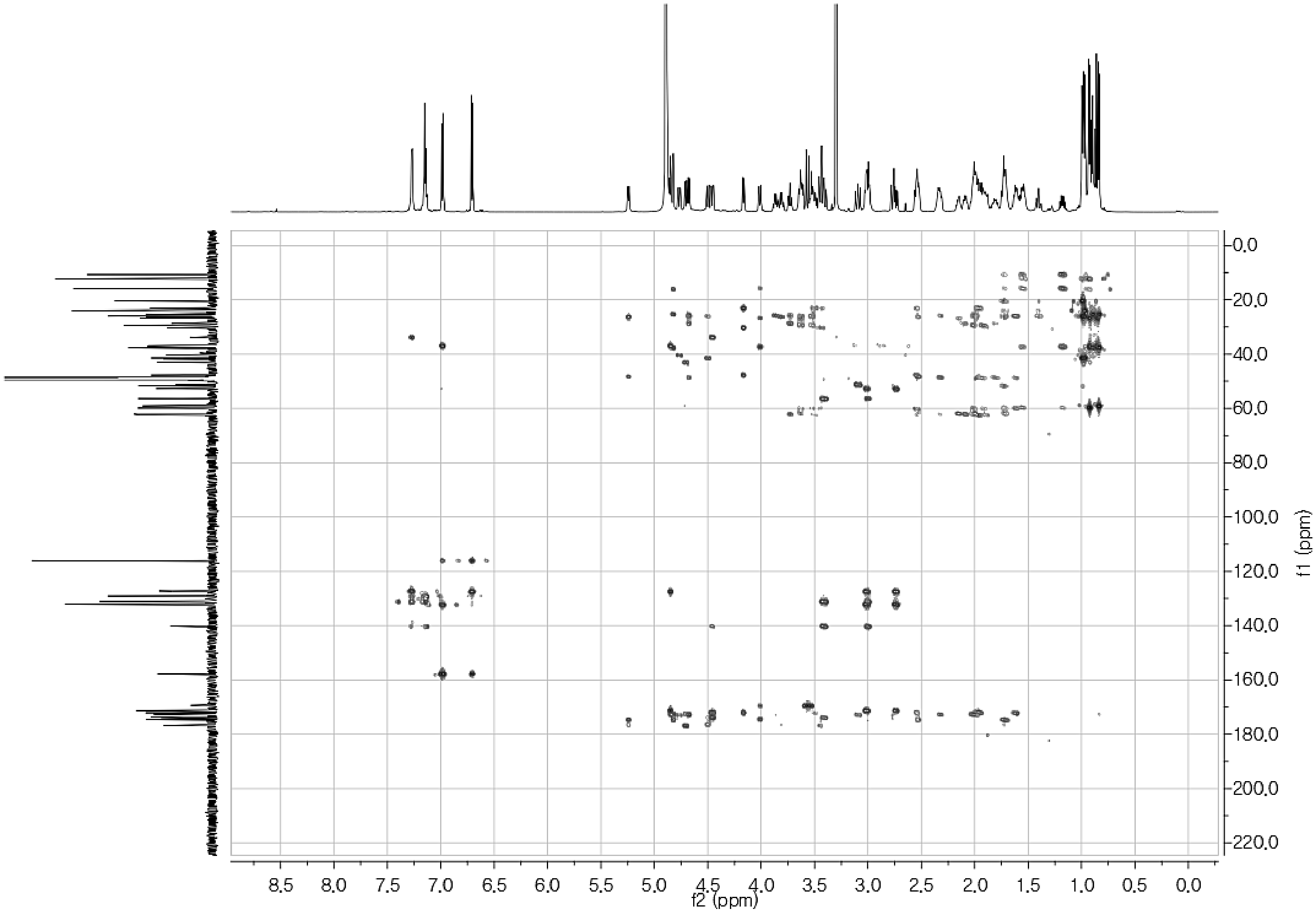

Figure S5. The TOCSY (600 MHz, MeOH-d_4) spectrum of compound **1**.

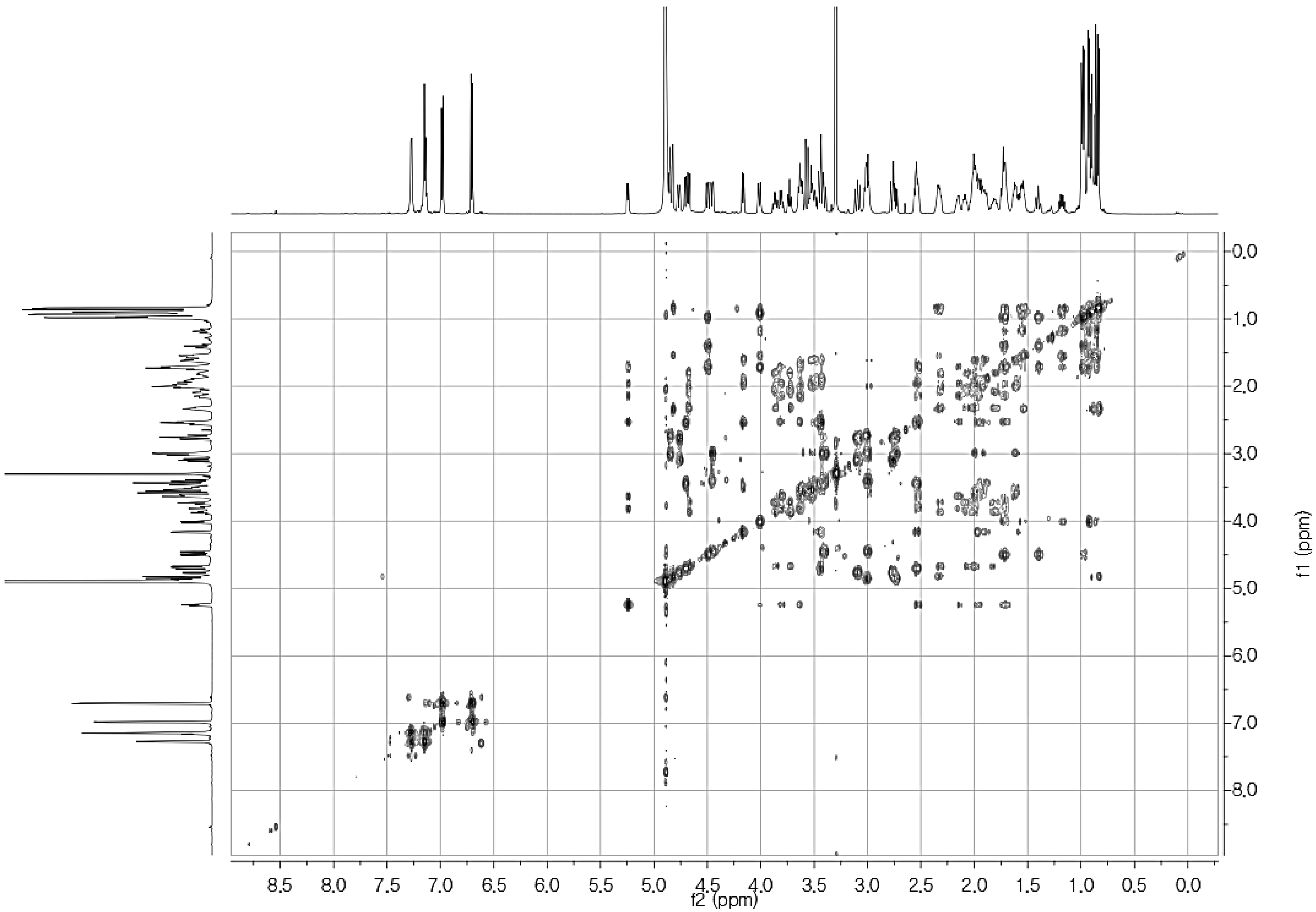

Figure S6. The COSY (600 MHz, MeOH-d_4) spectrum of compound **1**.

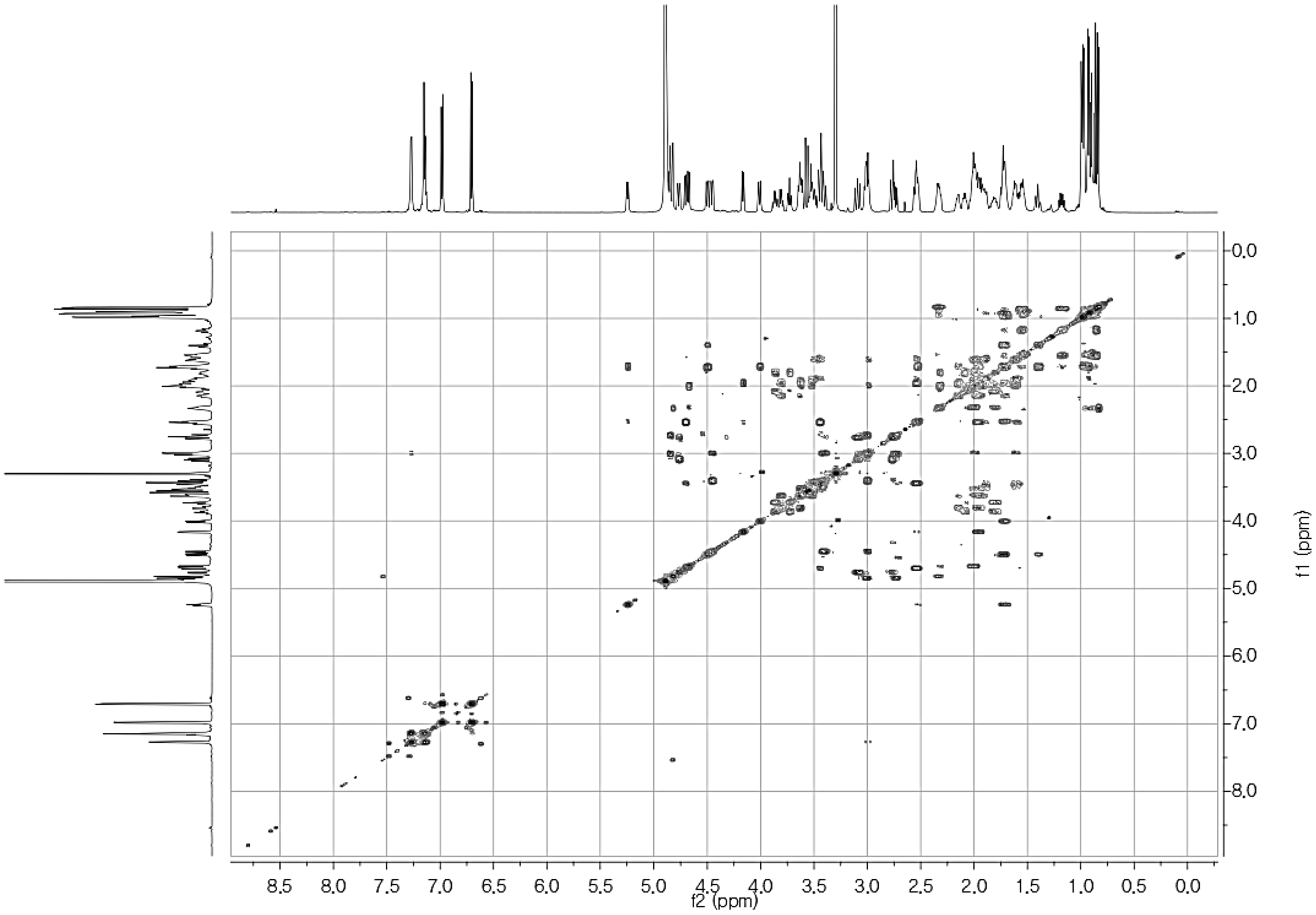

Figure S7. The ROESY (600 MHz, MeOH-d_4) spectrum of compound **1**.

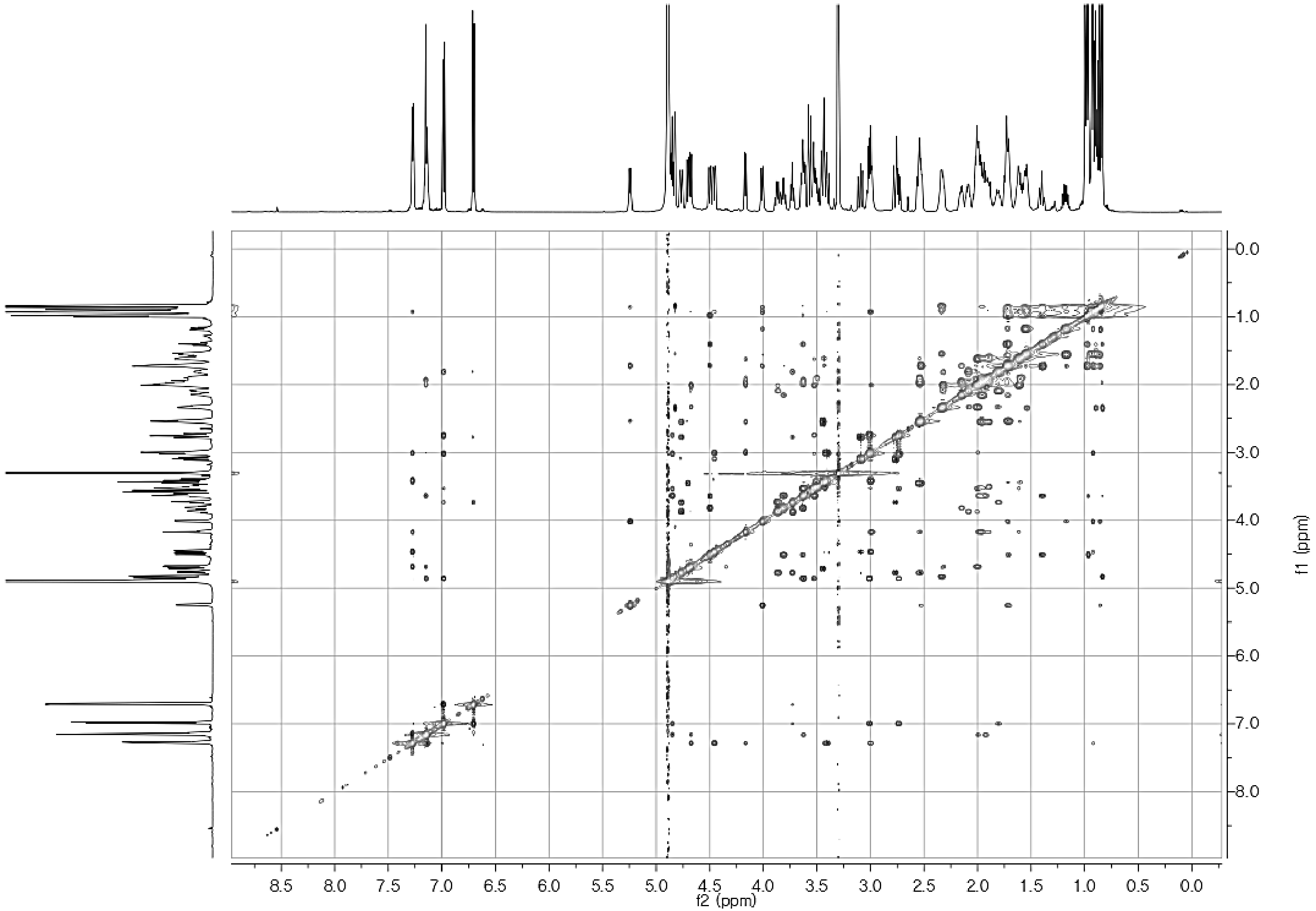

Figure S8. The ^{1}H NMR (600 MHz, MeOH-d_3) spectrum of compound **1**.

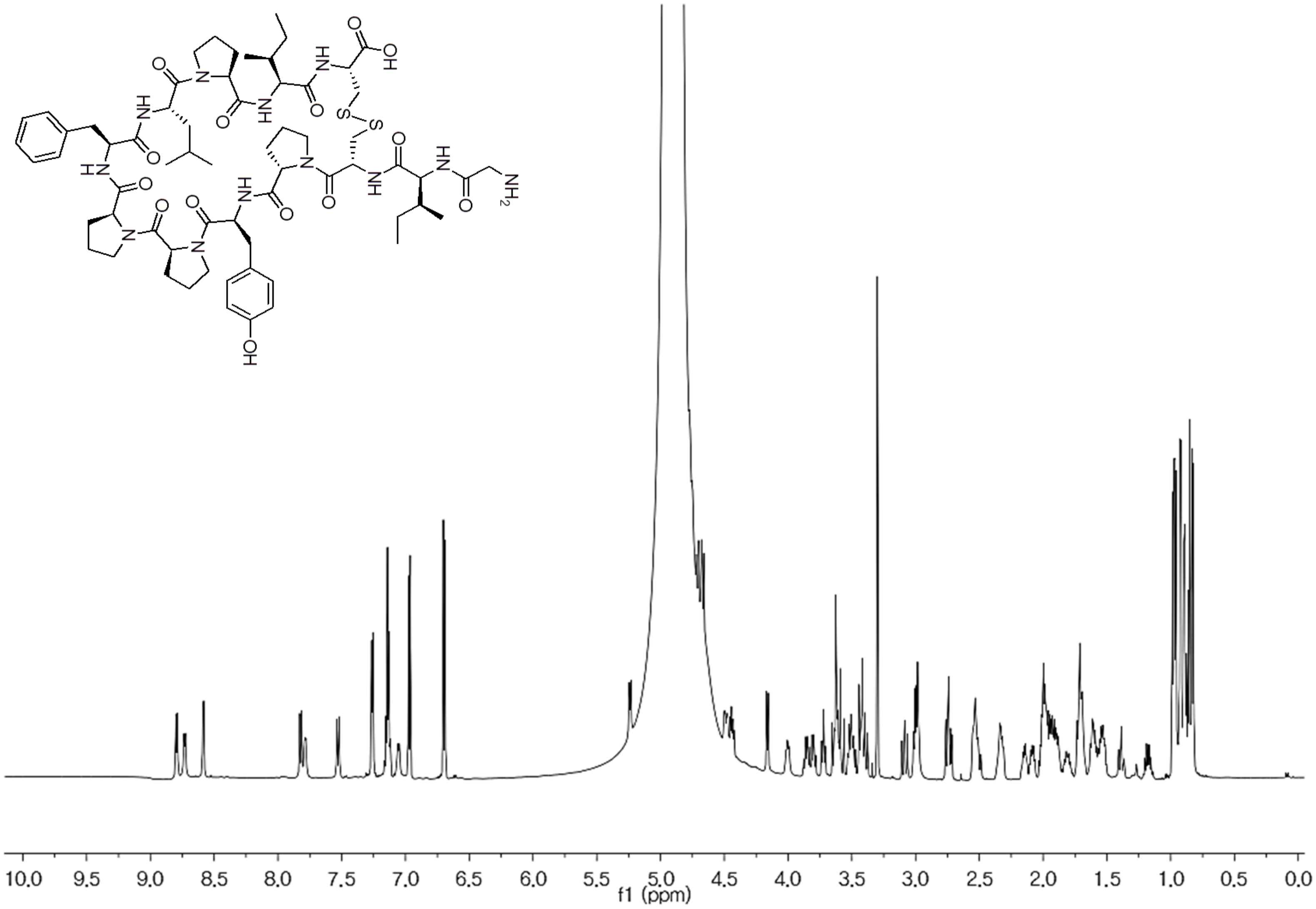

Figure S9. The *g*HMBC (600 MHz, MeOH-d_3) spectrum of compound **1**.

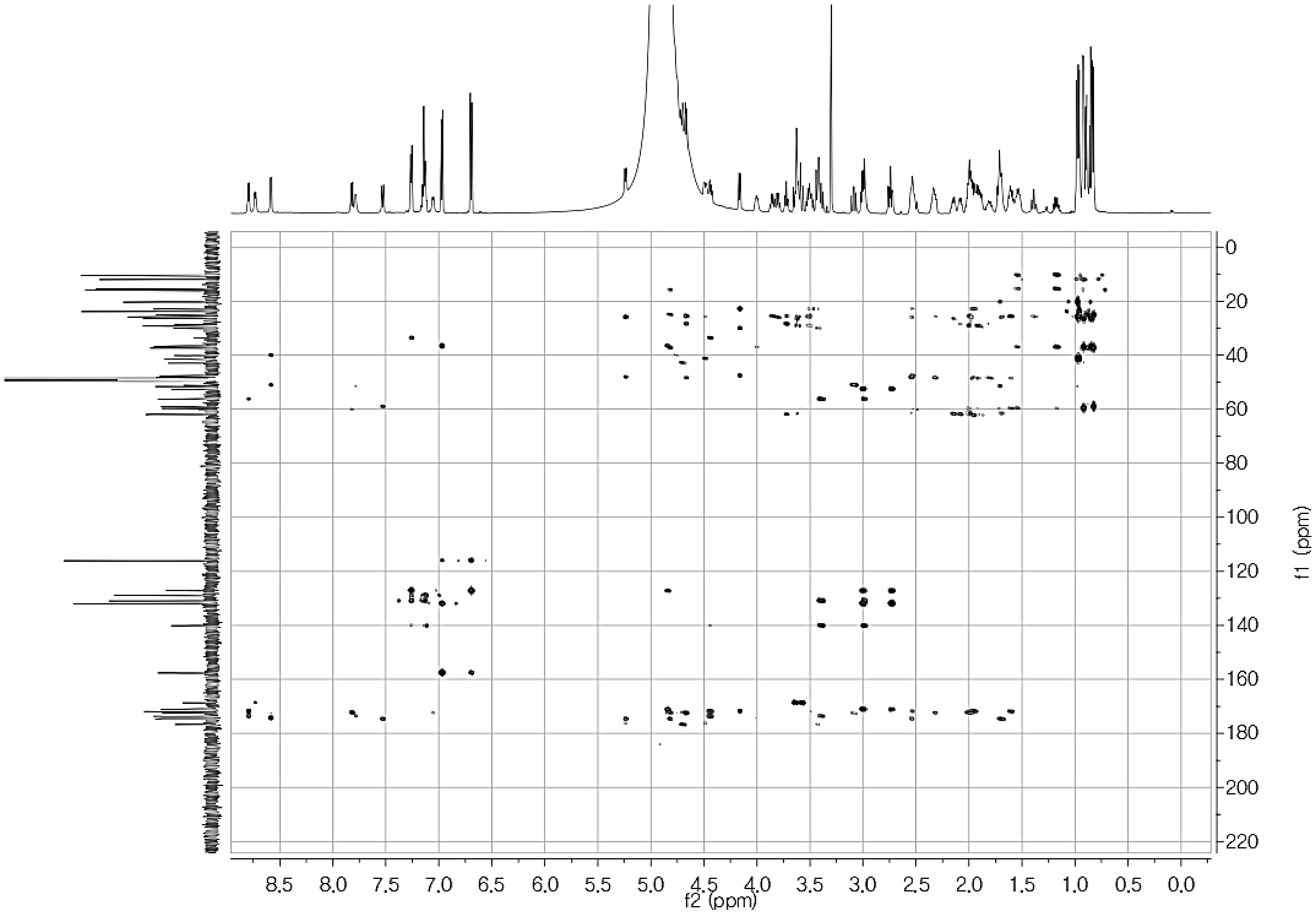

Figure S10. The TOCSY (600 MHz, MeOH-d_3) spectrum of compound **1**.

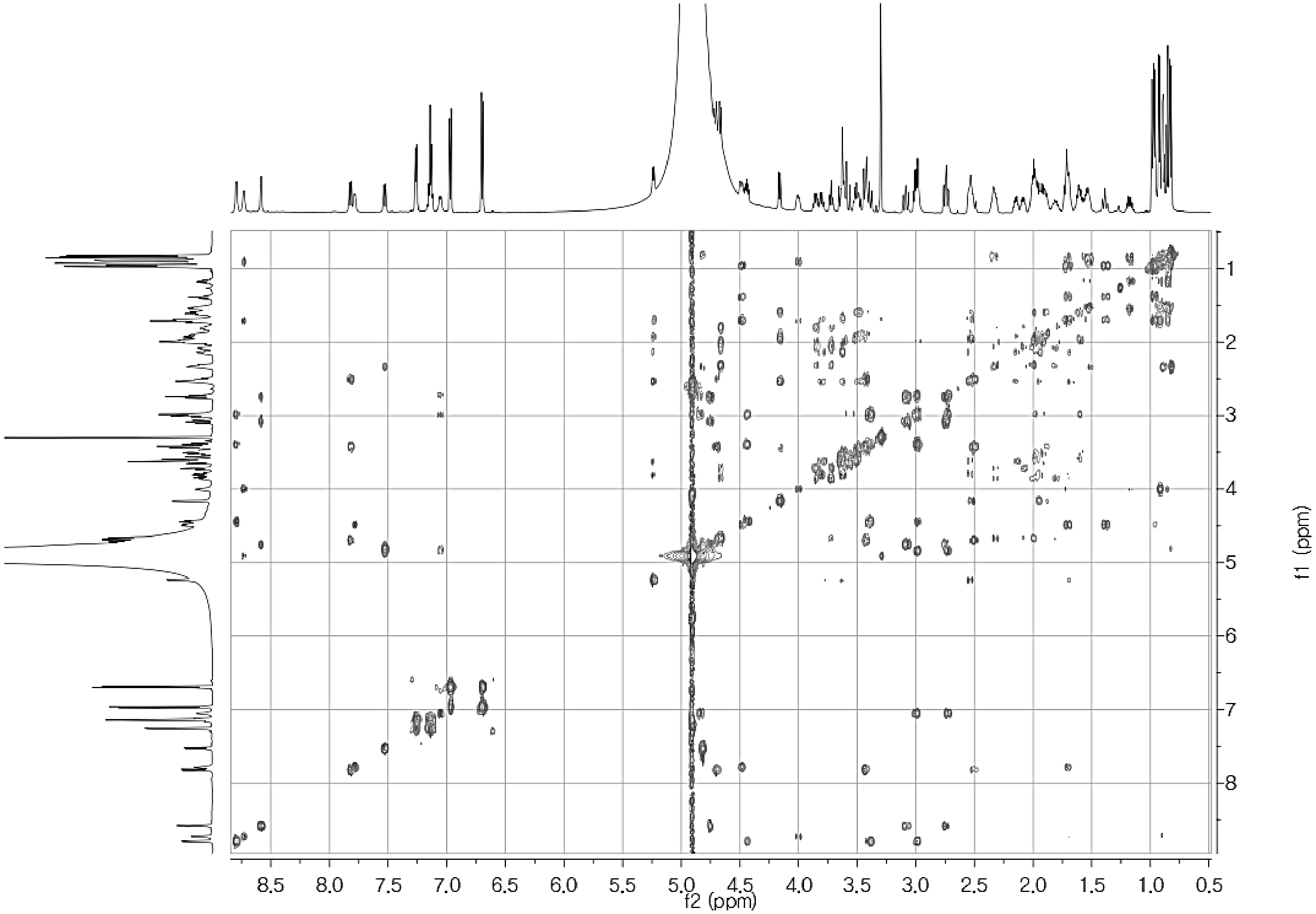

Figure S11. The COSY (600 MHz, MeOH-d_3) spectrum of compound **1**.

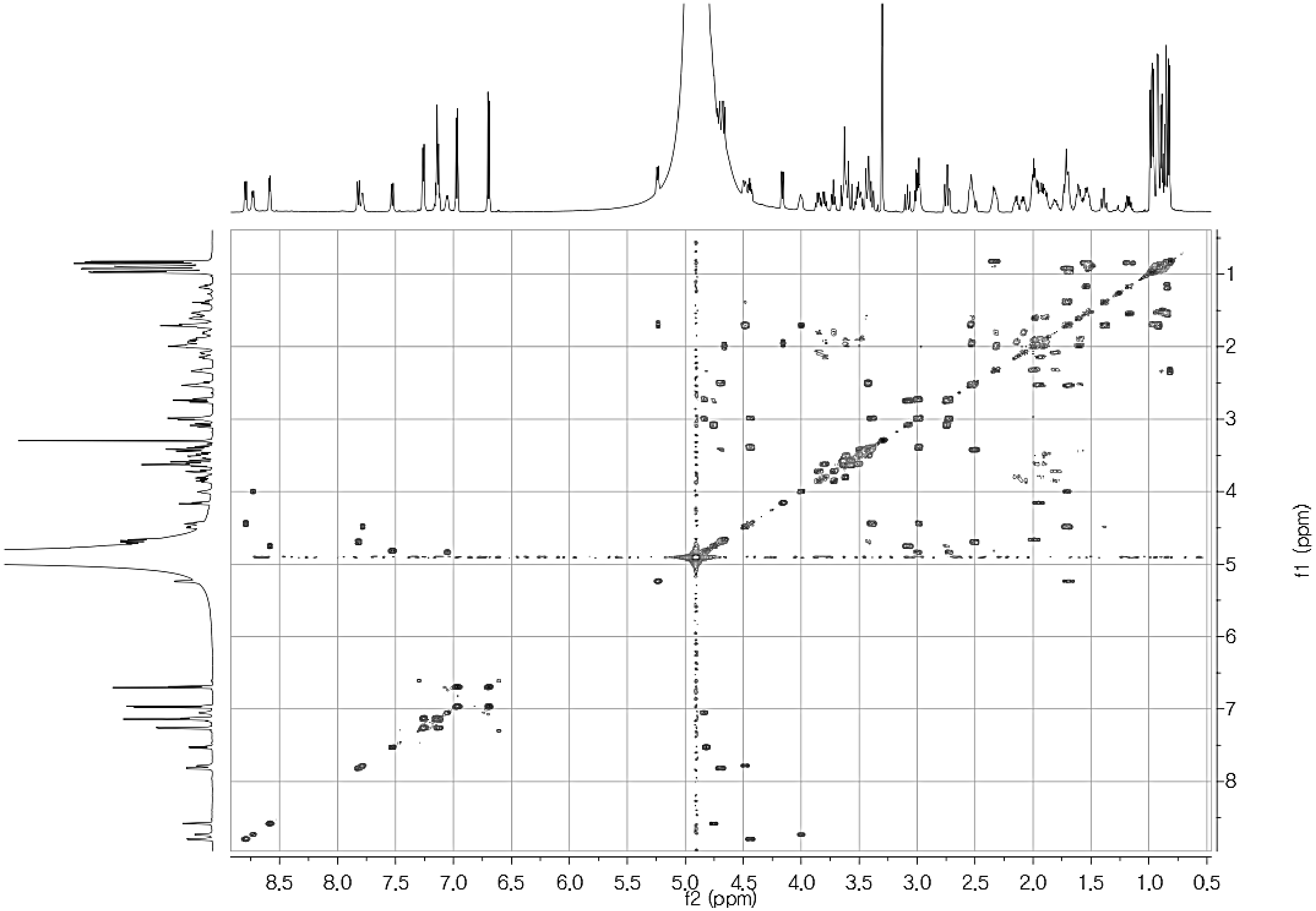

Figure S12. The ROESY (600 MHz, MeOH-d_3) spectrum of compound **1**.

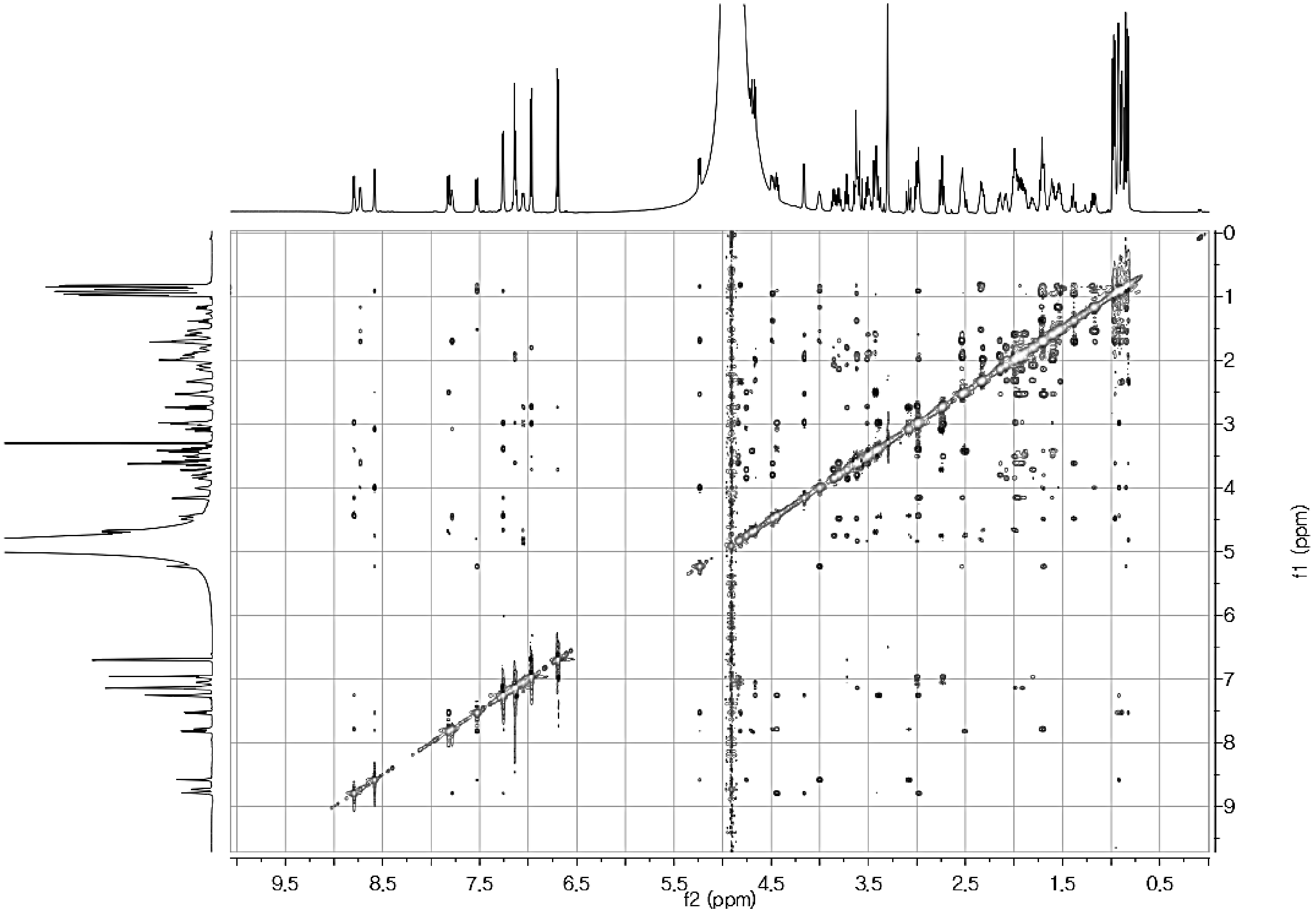

Figure S13. The ^{1}H NMR (600 MHz, MeOH-d_4) spectrum of compound **2**.

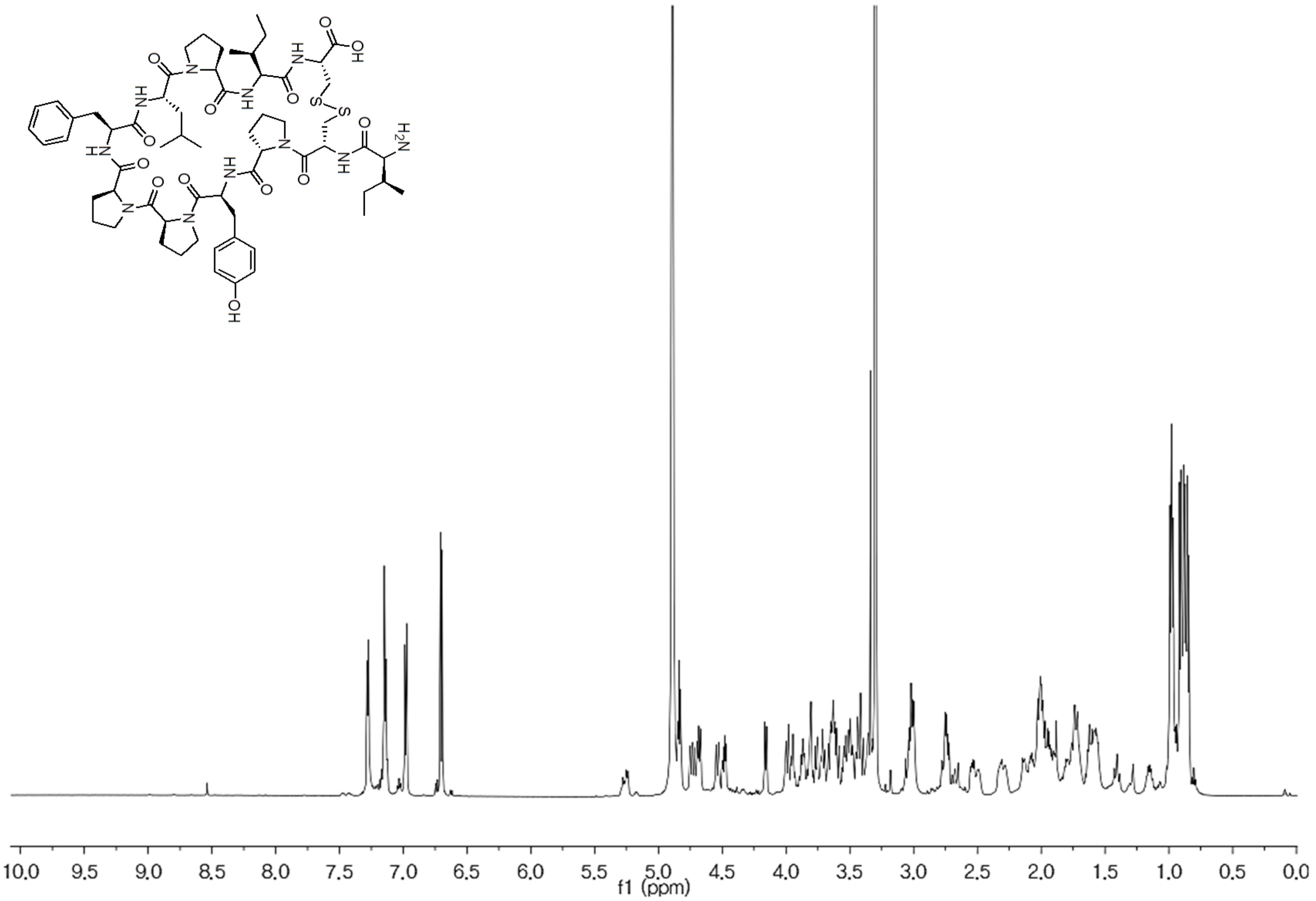

Figure S14. The ^{13}C NMR (150 MHz, MeOH-d_4) spectrum of compound **2**.

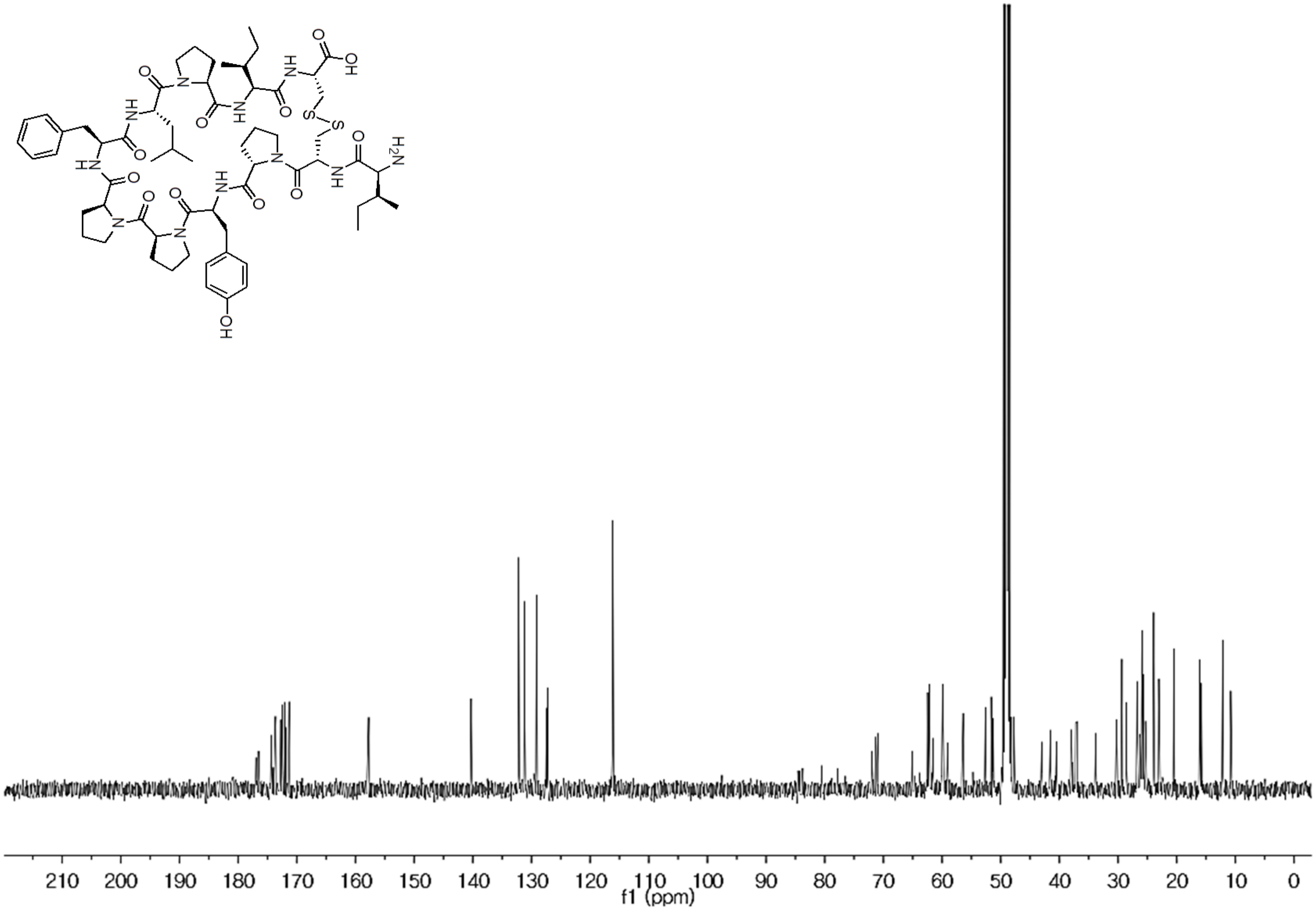

Figure S15. The *g*HSQC (600 MHz, MeOH-d_4) spectrum of compound **2**.

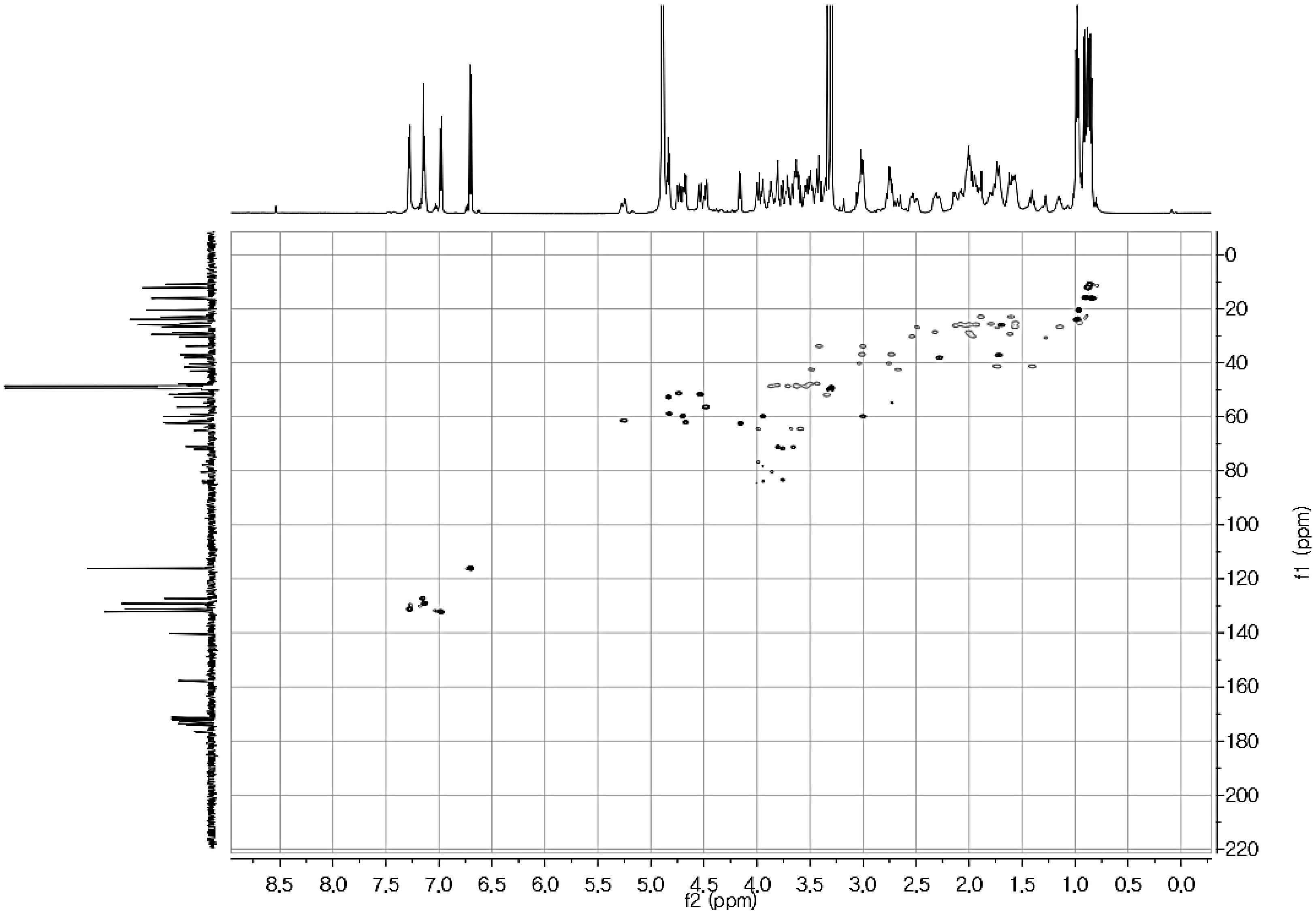

Figure S16. The *g*HMBC (600 MHz, MeOH-d_4) spectrum of compound **2**.

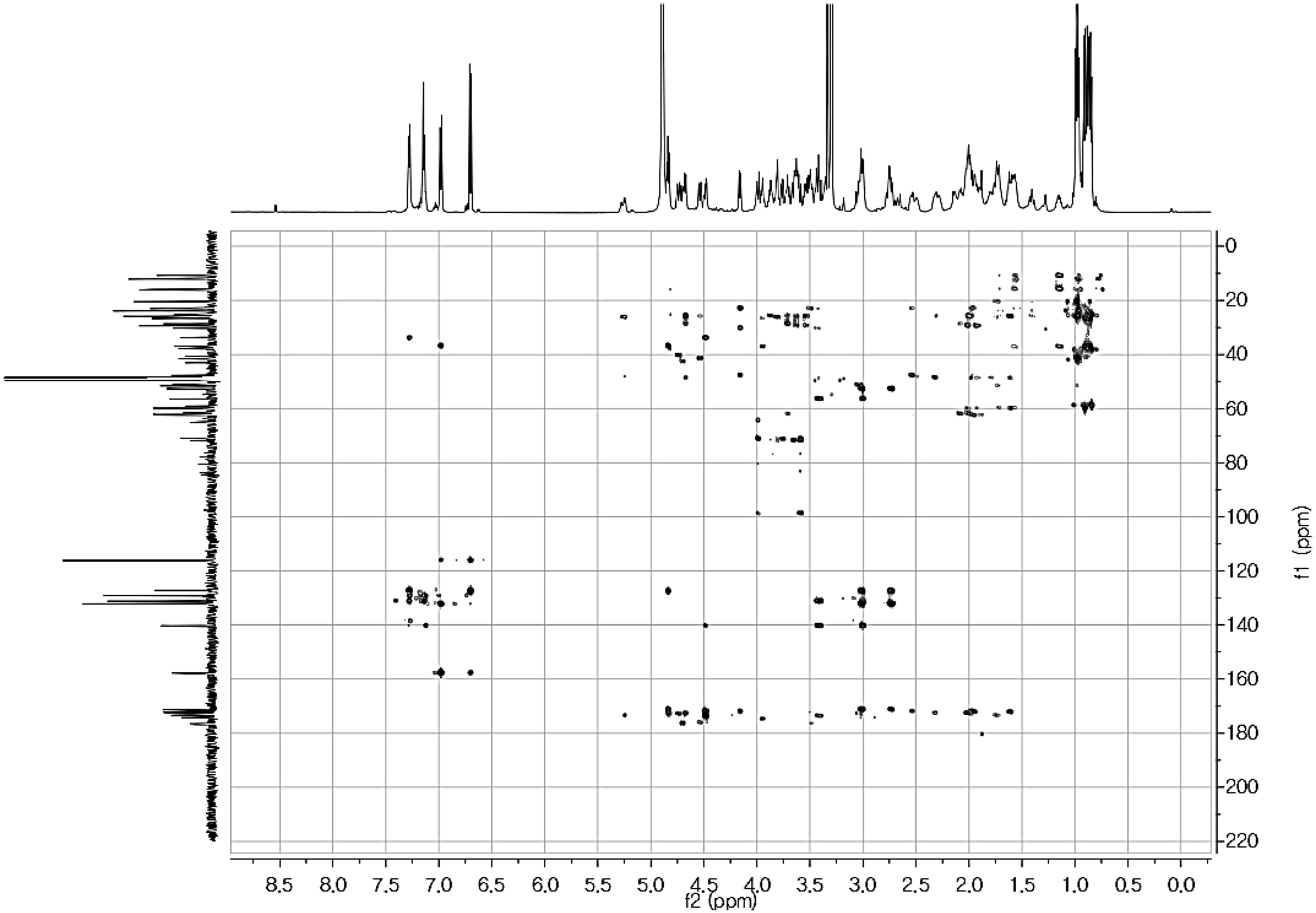

Figure S17. The TOCSY (600 MHz, MeOH-d_4) spectrum of compound **2**.

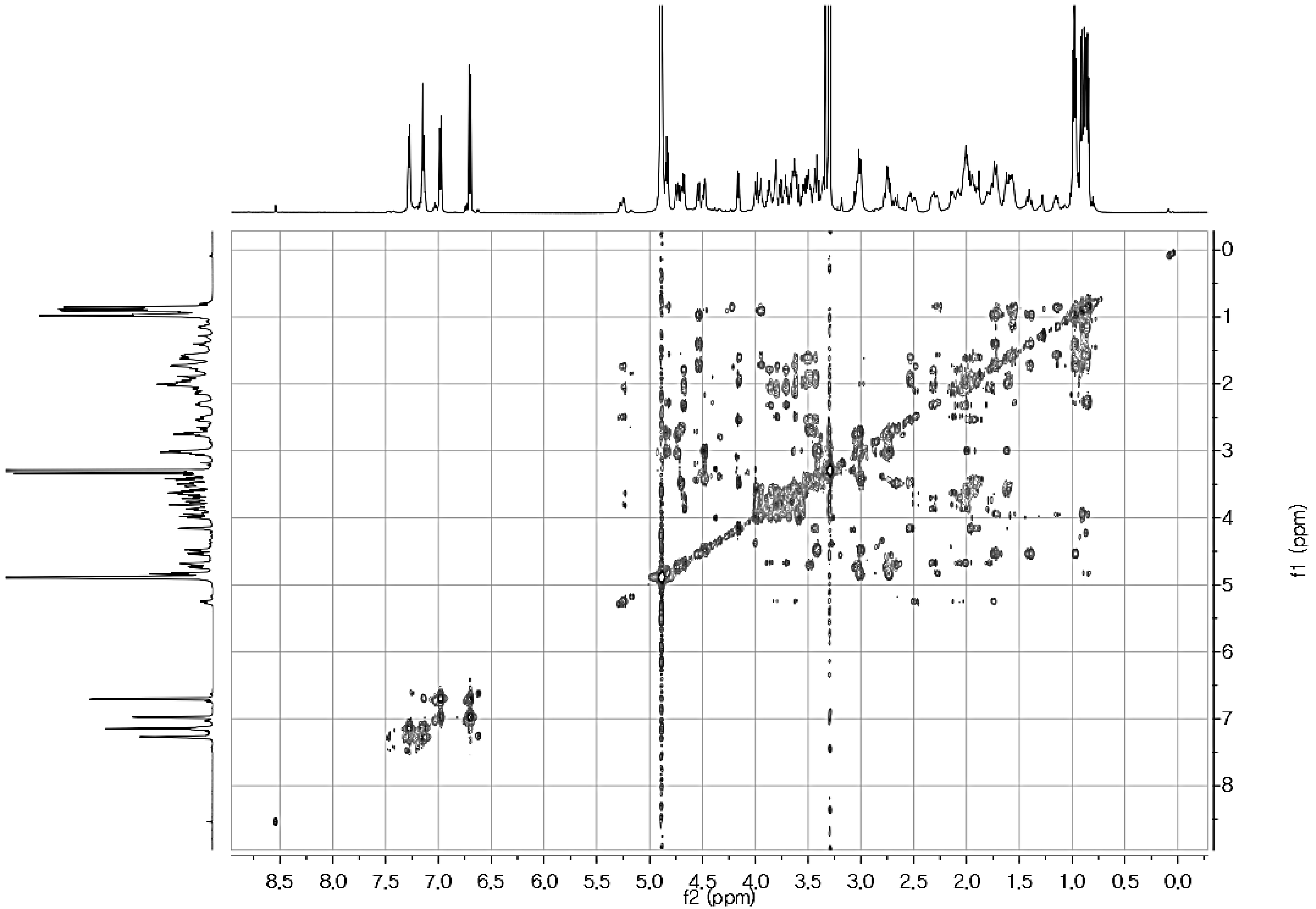

Figure S18. The COSY (600 MHz, MeOH-d_4) spectrum of compound **2**.

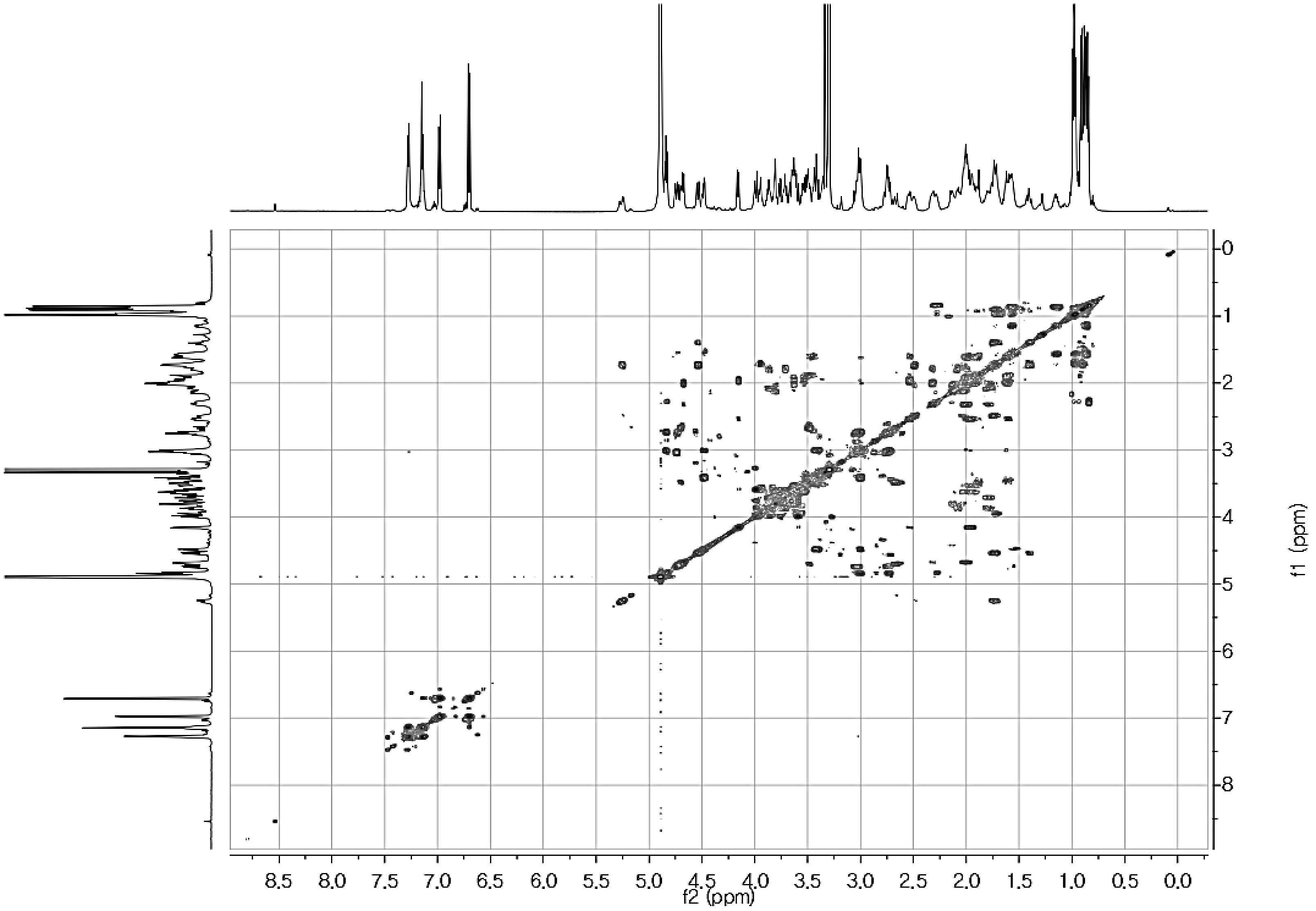

Figure S19. The ROESY (600 MHz, MeOH-d_4) spectrum of compound **2**.

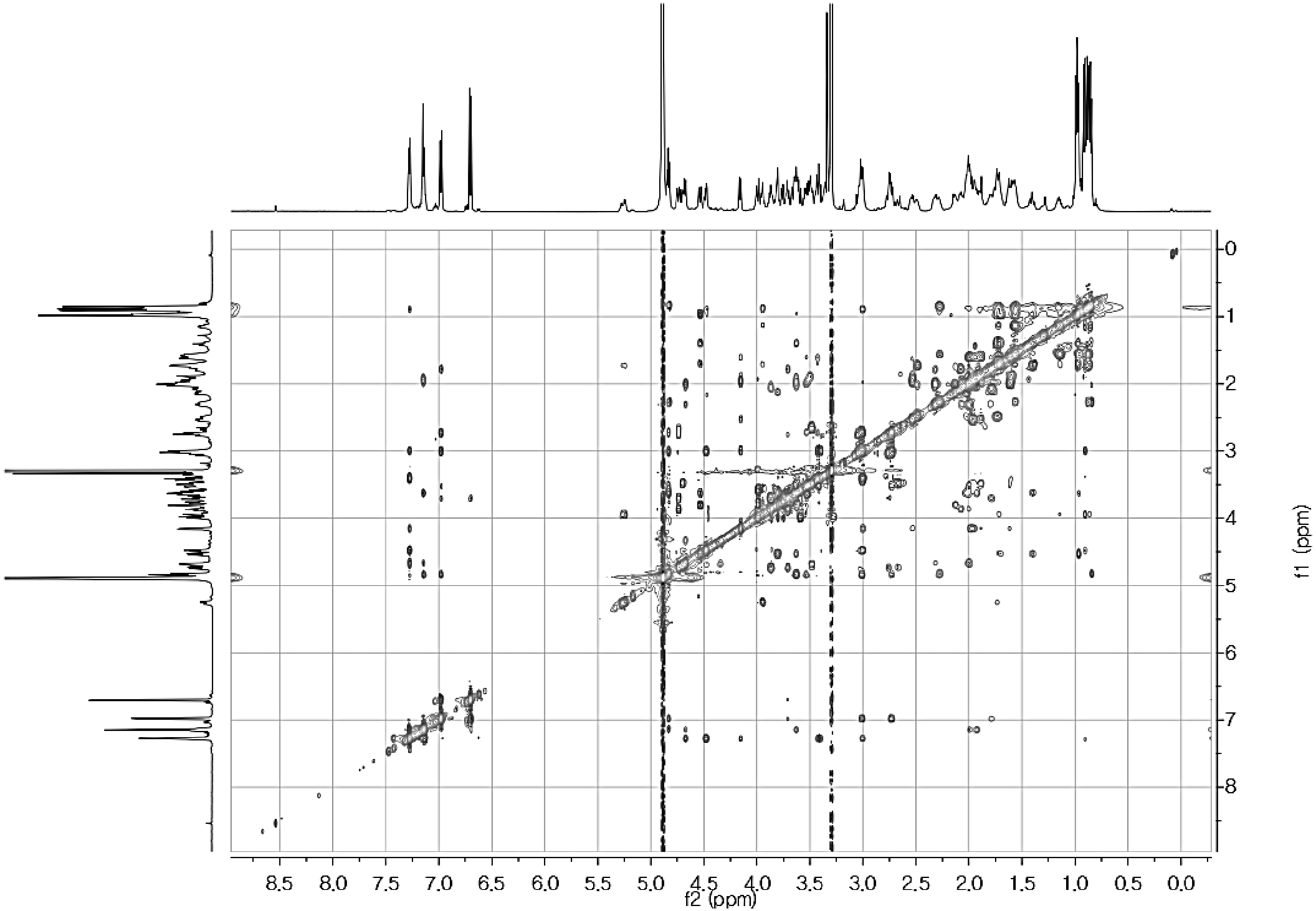

Figure S20. The ^{1}H NMR (600 MHz, MeOH-d_3) spectrum of compound **2**.

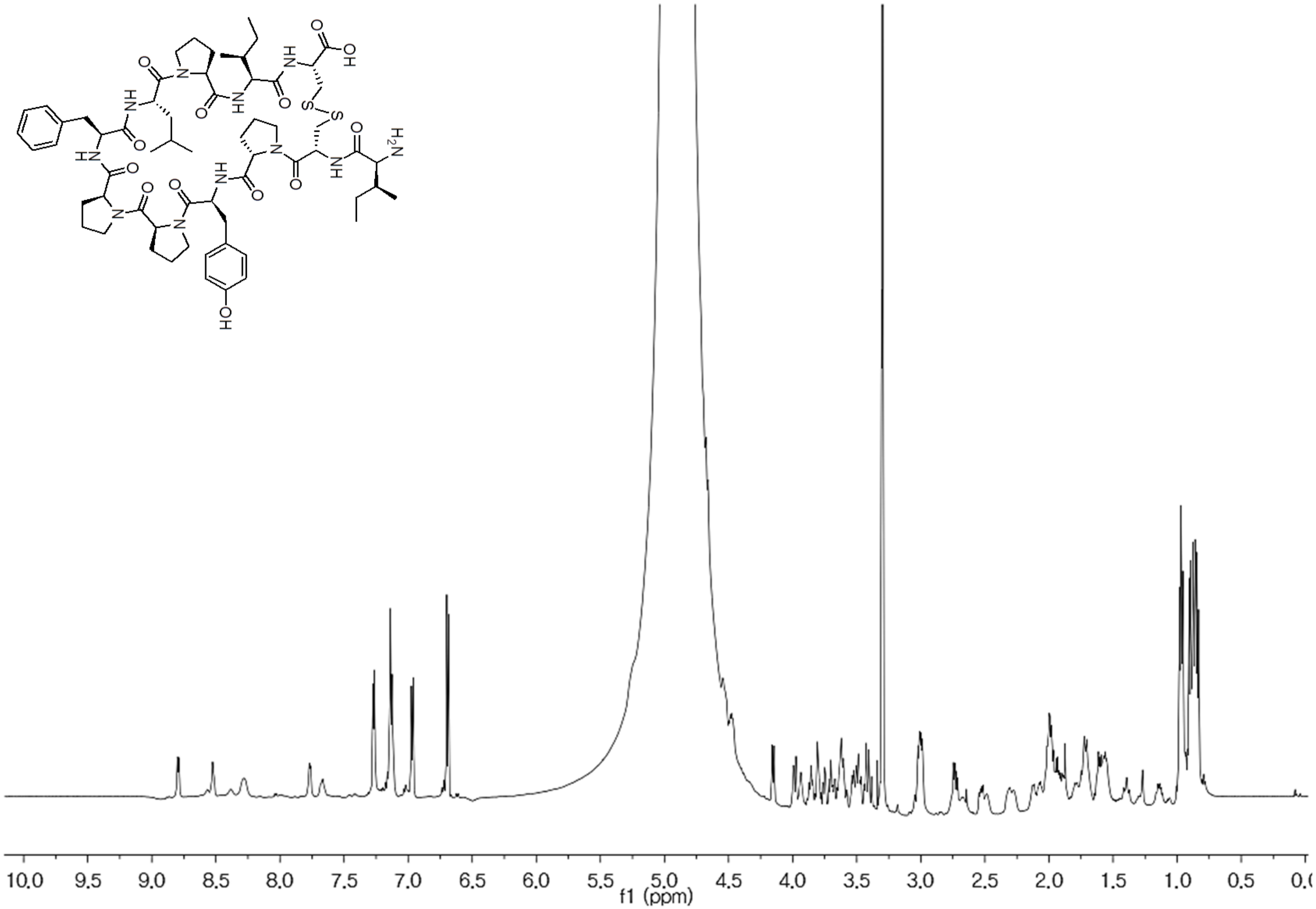

Figure S21. The *g*HMBC (600 MHz, MeOH-d_4) spectrum of compound **2**.

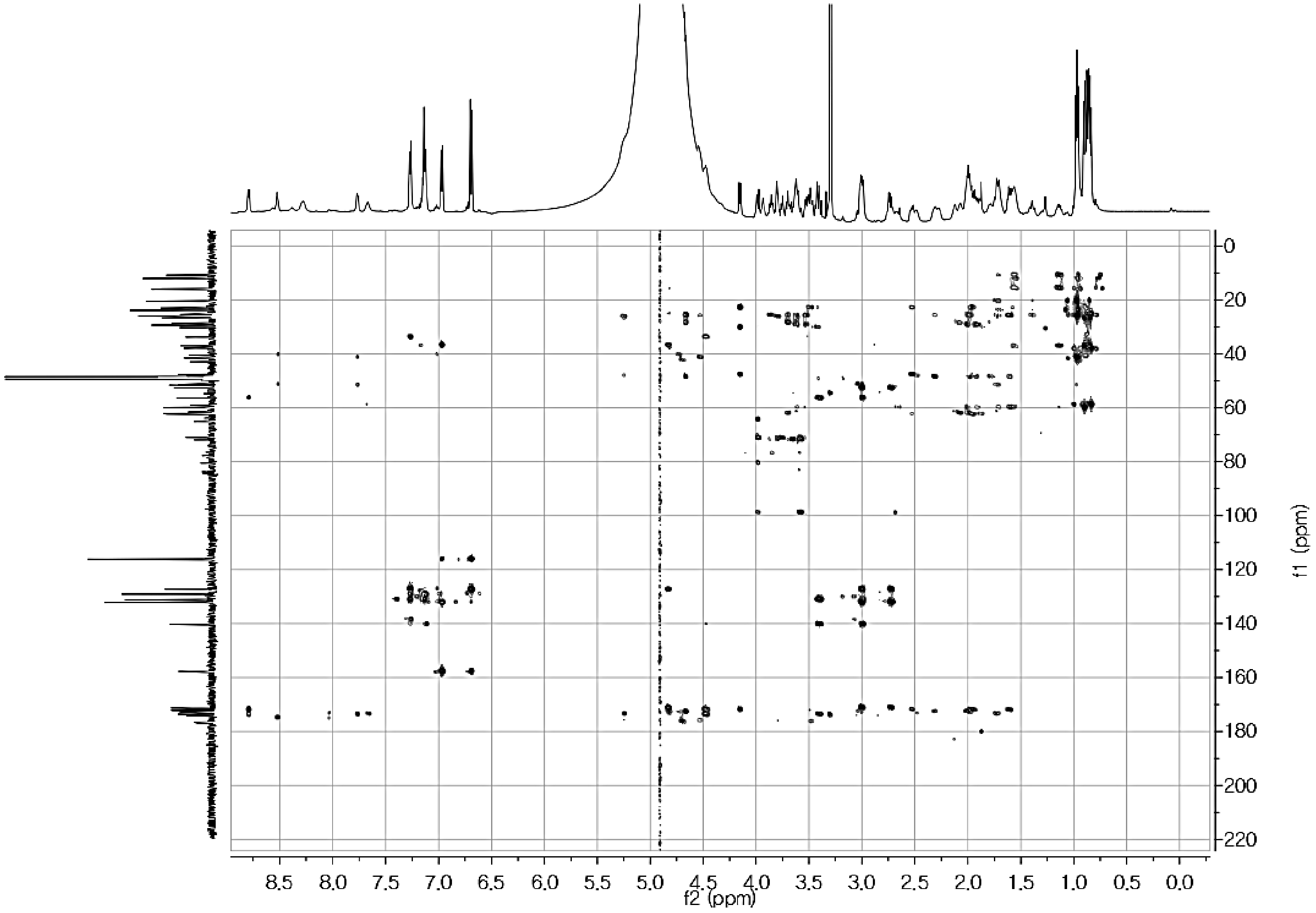

Figure S22. The TOCSY (600 MHz, MeOH-d_3) spectrum of compound **2**.

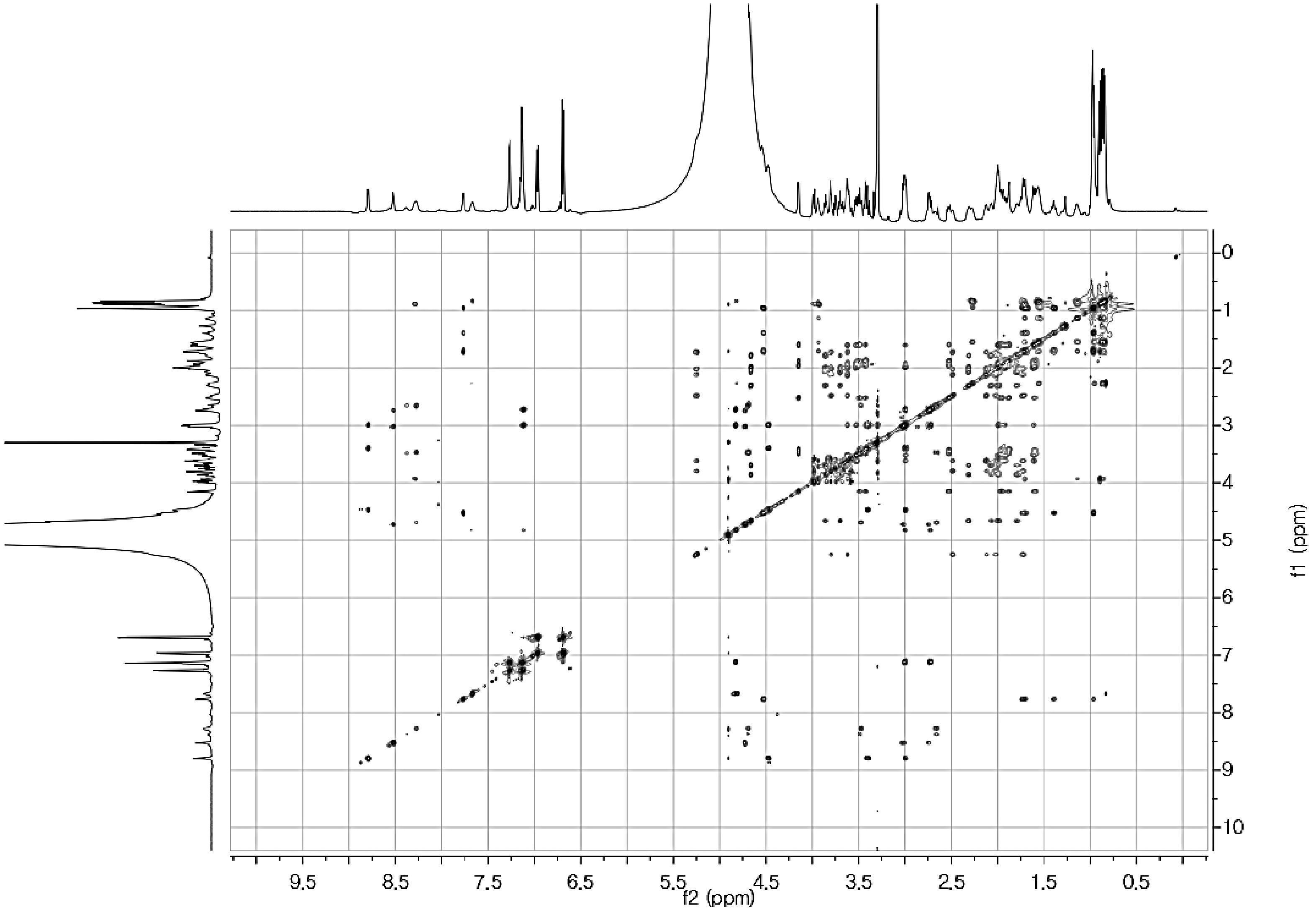

Figure S23. The COSY (600 MHz, MeOH-d_3) spectrum of compound **2**.

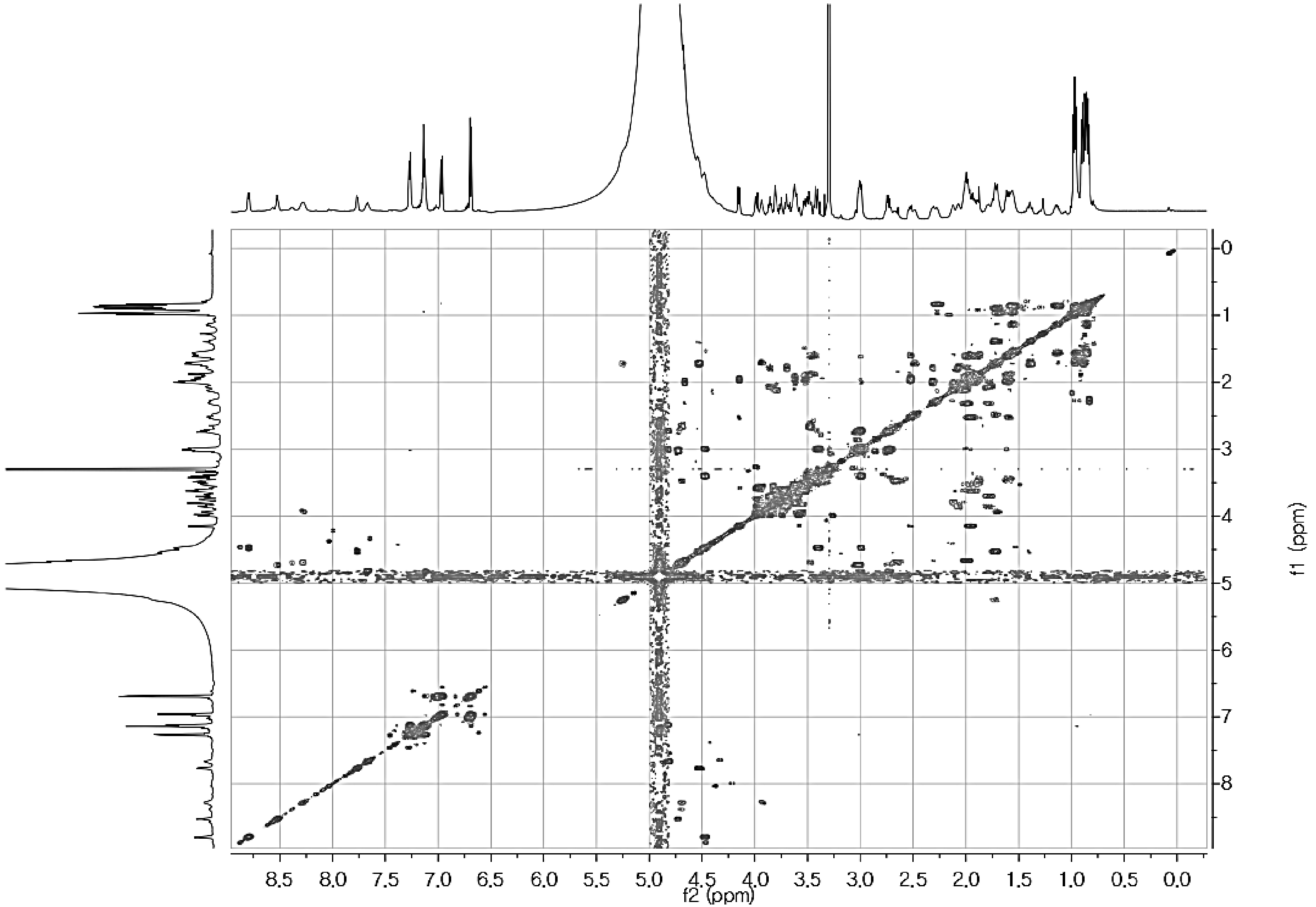

Figure S24. The ROESY (600 MHz, MeOH-d_3) spectrum of compound **2**.

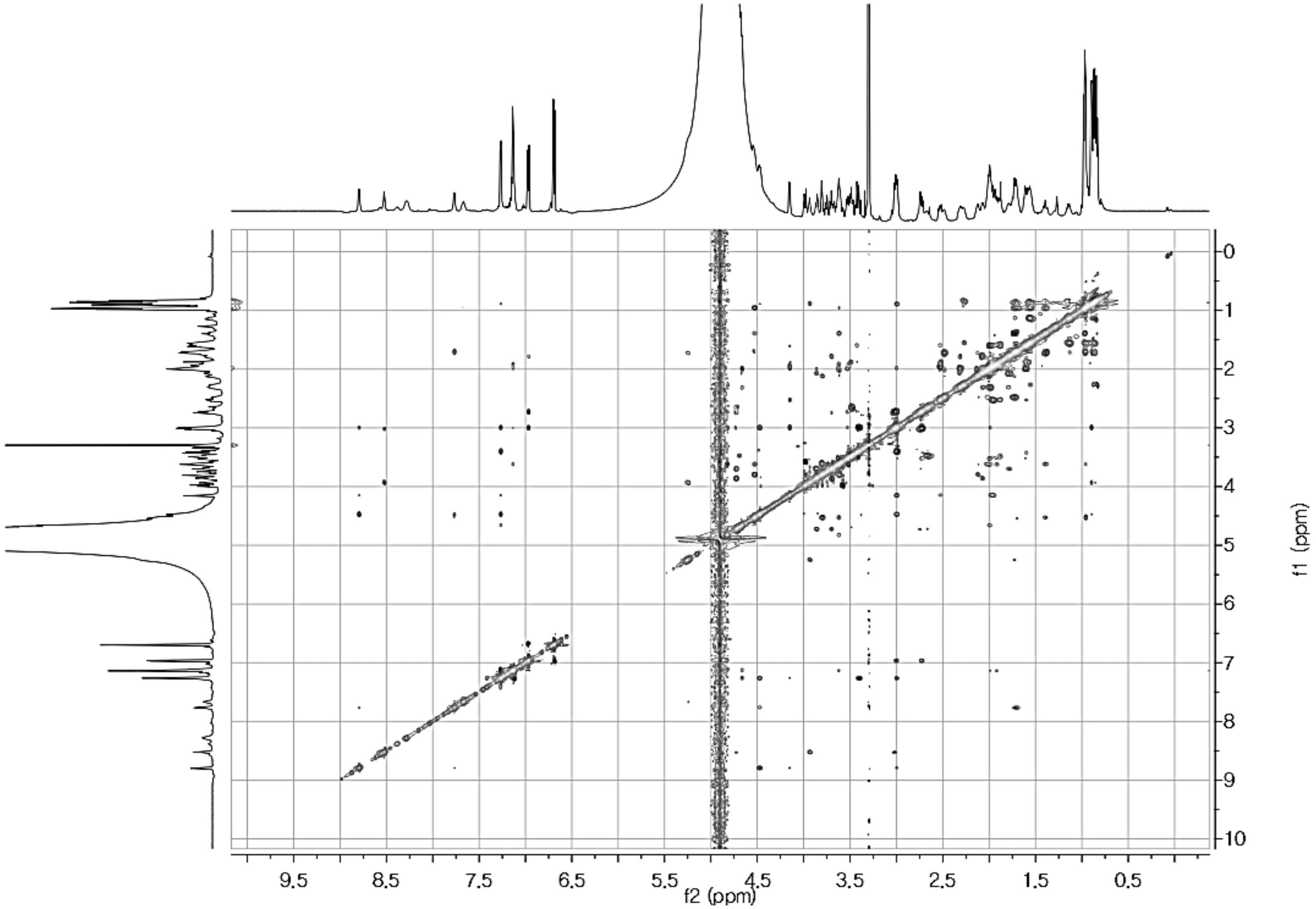

© 2014 by the authors; licensee MDPI, Basel, Switzerland. This article is an open access article distributed under the terms and conditions of the Creative Commons Attribution license (http://creativecommons.org/licenses/by/3.0/).
